# Coexpression of Three Odorant-Binding Protein Genes in the Foreleg Gustatory Sensilla of Swallowtail Butterfly Visualized by Multicolor FISH Analysis

**DOI:** 10.3389/finsc.2021.696179

**Published:** 2021-07-30

**Authors:** Atsushi Ugajin, Katsuhisa Ozaki

**Affiliations:** JT Biohistory Research Hall, Takatsuki, Japan

**Keywords:** butterfly, *Papilio xuthus*, host plant selection, odorant-binding protein, gustatory sensilla, fluorescence *in situ* hybridization

## Abstract

Lepidopteran insects are mostly monophagous or oligophagous. Female butterflies distinguish their host plants by detecting a combination of specific phytochemicals through the gustatory sensilla densely distributed on their foreleg tarsi, thereby ensuring oviposition on appropriate host plants. In this study, to gain insight into the molecular mechanism underlying host plant recognition by the gustatory sensilla, using Asian swallowtail, *Papilio xuthus*, we focused on a family of small soluble ligand-binding molecules, odorant-binding proteins (OBPs), and found that three OBP genes showed enriched expression in the foreleg tarsus. Multicolor fluorescence *in situ* hybridization analyses demonstrated the coexpression of these three OBP genes at the bases of the foreleg gustatory sensilla. Further analyses on other appendages revealed that *PxutOBP3* was exclusively expressed in the tissues which could have direct contact with the leaf surface, suggesting that this OBP gene specifically plays an important role in phytochemicals perception.

## Introduction

Most phytophagous (plant-feeding) insects utilize a limited range of host plants (1, 2). For these monophagous and oligophagous insect species, precise discrimination of right host plants is fundamentally important (3, 4). In Lepidoptera, because of low mobility of larvae, host plant selection is mainly executed by female adults during oviposition (5). Before egg laying, female butterflies quickly drum their forelegs on the leaf surface to examine whether a plant is the suitable host species for larvae by detecting chemicals secreted from the leaves through chemosensilla densely distributed on the ventral surface of the foreleg tarsi (6, 7).

Asian swallowtail, *Papilio xuthus*, belongs to the family of Papilionidae. *P. xuthus* larvae feed exclusively on Rutaceae plants, whereas adults suck floral nectar and no longer feed on leaves (8, 9). A complete set of oviposition stimulants for *P. xuthus*, composed of 10 compounds in the Rutaceae leaves, has been identified (10). We previously demonstrated that the foreleg gustatory sensilla housed at least three types of gustatory receptor neurons which were tuned for five oviposition stimulants (11), and identified *PxutGr1* as a receptor gene for one of the oviposition stimulants, synephrine (12). Importantly, however, it has been argued that not only chemoreceptors but also some soluble ligand-binding proteins cooperatively play a crucial role in chemoreception (i.e., olfaction and gustation) (13, 14).

The family of odorant-binding proteins (OBPs) is a representative of the soluble ligand-binding molecules (15–17). Insect OBPs are small (~15 kDa) proteins and generally consist of 130–150 amino acids (15). A structural feature of OBPs is a set of six cysteine residues that form three disulfide bridges providing stable globular conformations (15). According to the conserved cysteine patterns, OBPs are categorized into three classes: classic, minus-C, and plus-C OBPs (15–17). Most of the OBP genes are expressed in support cells located at the bases of chemosensory sensilla, especially olfactory sensilla, and synthesized proteins are localized in the sensillar lymph (16, 17). Functional analysis proposed several roles of OBPs in chemoreception, for example, transport of hydrophobic chemicals through the sensillar lymph to chemoreceptors, protection of ligands from degradative enzymes, and filtering chemicals (16, 17). Some OBPs of *Drosophila melanogaster* were reported to contribute to taste perception (18–20). Notably, altered expression level of OBPs in the leg gustatory sensilla affected to taste perception and host plant preference in a close relative of *D. melanogaster* (21–23).

There has been limited information on *P. xuthus* OBPs. We previously constructed EST libraries from *P. xuthus* females but obtained only three OBP sequences (24). In this study, we performed a genome-wide search of *P. xuthus* OBP genes, and then identified additional 41 OBP genes, and found that 3 out of in total 44 OBP genes showed enriched expression patterns in the foreleg tarsus. Subsequent multicolor fluorescence *in situ* hybridization (FISH) analyses revealed coexpression of these three OBP genes in identical support cells at the bases of the foreleg gustatory sensilla.

## Materials and Methods

### Animals

*P. xuthus* used in this study were laboratory-raised summer forms. Adult females were collected at Takatsuki, Osaka, Japan, and allowed to lay eggs on leaves of *Citrus unshu* in the laboratory at 25°C under light/dark photoperiods of 16 and 8 h, respectively. Larvae were reared on artificial diet (mixture of Insecta F-Ii (Nosan, Kanagawa, Japan) and powder of dried *Zanthoxylum ailanthoides* leaves).

### RNA-Sequencing and *De novo* Assembly

We collected three pairs of forelegs from 0-, 1-, 3-, and 5-day-old females. Also, a pair of antennae from a 0-day-old female was also collected. Total RNA samples were extracted using QuickGene RNA tissue kit SII (Kurabo, Osaka, Japan). cDNA library preparation was carried out using TruSeq RNA Sample Prep Kit v2 (Illumina, CA, USA) following the manufacturer's protocol with a modification of the incubation time for RNA fragmentation. To obtain longer fragments, we set the incubation time to 1 min. RNA-sequencing runs were performed using MiSeq system with MiSeq sequencing kit v3 (Illumina). Raw RNA-sequencing data have been deposited in the DNA Data Bank of Japan (DDBJ) Sequence Data Archive under accession numbers DRA011862 and DRA011865. Sequenced reads were assembled using Trinity 2.0.6 and 2.8.4 for antenna samples and foreleg samples, respectively, with Quality Trimming Options by Trimmomatic (LEADING: 10; TRAILING: 10; SLIDINGWINDOW: 4:20; MINLEN: 150). Kmer_size parameter was set at 32. Coding regions and amino acid sequences of the assembled contigs were predicted using TransDecoder.

### Identification of PxutOBP Genes

To obtain candidate OBP genes, we performed BLASTp searches in both the genome database of *P. xuthus* and our assembled contigs (*e*-value > 0.01), using each of the full set of individual *Danaus plexippus, Heliconius melpomene*, and *Manduca sexta* OBPs reported previously (25) as queries (Supplementary Data Sheet 1). We also performed a HMMER search in our assembled contigs with the Pfam database and collected contigs that could encode the proteins classified as PBP_GOBP (*e*-value > 1e-10). Possible OBP-encoding contigs which found only by HMMER search were evaluated through further BLASTp searches against the NCBI non-redundant protein database (nr) and those homologous to OBPs of other insect species (*e*-value > 0.05) were additionally considered as candidate OBP genes. Subsequently, the deduced amino acid sequences of the candidate OBP genes were aligned by MUSCLE program in MEGA6 software (26) and confirmed the position of conserved cysteines.

### Phylogenetic Analysis

To examine the phylogenetic relationship between PxutOBPs and other lepidopteran OBPs, we performed a phylogenetic tree construction using amino acid sequences of OBPs identified in *D. plexippus, H. melpomene, M. sexta, Bombyx mori*, and *Vanessa cardui* (Supplementary Data Sheet 1). OBP sequences of *V. cardui* was kindly provided by Mr. Hiromu C. Suzuki (27). After the multiple alignments, the neighbor-joining (NJ) tree was generated using MEGA with 1,000 rounds of bootstrapping (*p*-distance, pairwise deletion; otherwise default settings).

### Quantitative RT-PCR

After eclosion, each adult female was individually kept in a translucent plastic cup under *ad libitum* feeding on Pocari Sweat (Otsuka Pharmaceutical, Tokyo, Japan). If needed, mating was performed artificially (hand-pairing) a day after eclosion. Note that all of the adult females had never touched any plant leaves. A previous electrophysiological study reported essentially no differences in response patterns of the foreleg gustatory sensilla varying in age, at least from 0 to 8 days after eclosion (11). To avoid the deficit of appendages during keeping in a small cup, we mainly used adults 0 day after eclosion. Appendages were collected by forceps into 1.5 ml tubes floating on liquid nitrogen and stored at −80°C until use. Total RNA samples were isolated using RNeasy Micro Kit (Qiagen, The Netherland) and then reverse-transcribed using PrimeScript RT Reagent Kit with gDNA Eraser (Takara, Shiga, Japan), which eliminated potentially contaminated genomic DNA. Quantitative reverse transcription-polymerase chain reaction (qRT-PCR) was performed using SYBR Premix Ex Taq II (Tli RNase H Plus) (Takara) and Thermal Cycler Dice Real Time System II (Takara) in accordance with the manufacturer's protocol and with gene-specific primers listed in Supplementary Table 1. All primer sets were designed to amplify 100–150 bp fragments, and standard curves were prepared using six points with progressive quantities of PCR amplicons (1 × 10^−1^ to 1 × 10^−6^ pg/μl). Relative expression levels of each gene were calculated using the expression value of *ribosomal protein L32* (*Rpl32*). Statistical analyses were conducted using Student's *t*-test, Tukey–Kramer's test, or Dunnett's test with STATCEL2 (OMS, Saitama, Japan).

### Fluorescence *in situ* Hybridization

Riboprobes were prepared by *in vitro* transcription using PCR products as a template (Supplementary Table 2). Digoxigenin (DIG)- and fluorescein (FLU)-labeled riboprobes were synthesized using RNA labeling kits (Roche, Basel, Switzerland). To synthesize 2,4-dinitrophenyl (DNP)-labeled probes, we prepared a 10× solution [3.5 mM DNP-11-UTP (PerkinElmer, MA, USA), 6.5 mM UTP, 10 mM ATP, CTP, and GTP] and performed *in vitro* transcription as well as the other probes. Synthesized products were purified by LiCl precipitation.

Tissues were embedded in Tissue-Tek O.C.T. Compound (Sakura Finetek, Tokyo, Japan), immediately frozen in a deep freezer, and stored at −80°C until use. 10 μm fresh-frozen sections were prepared using a cryostat (OT/FAS/EC/MR/Z, Bright Instrument Company, UK) set at −18°C. Sections were collected on APS-coated microscope slides (Matsunami Glass Ind, Osaka, Japan) using wooden toothpicks. After overnight air-drying, sections were fixed in 4% paraformaldehyde in 0.1 M phosphate buffer (PB) overnight at 4°C, treated with 12.5 μg/ml proteinase K (29442-14, Nacalai, Kyoto, Japan) for 15 min and then with 0.2 M HCl for 10 min, followed by acetylation solution [0.25% acetic anhydride, 0.1 M pH 8.0 Triethanolamine hydrochloride (T1502, Sigma-Aldrich, MO, USA)] for 10 min at room temperature. Slides were rinsed with PB between each step. After dehydration through a series of ethanol solutions (70, 80, 90, and 100%), sections were hybridized with the riboprobes overnight at 60°C. The riboprobes were diluted in hybridization buffer (50% formamide, 10 mM Tris-HCl (pH 7.6), 200 μg/ml yeast tRNA (15401-011, Thermo Fisher Scientific, MA, USA), 50 μg/ml heparin, 1× Denhardt's solution, 100 mg/ml sodium dextran sulfate, 0.6 M NaCl, 0.25% SDS, 1 mM EDTA) at a concentration of 1 μl/ml, heat-denatured at 85°C for 5 min, and then added to each slide. A strip of Parafilm (Bemis, IL, USA) was placed on top and slides were incubated in a box moisturized with 50% formamide at 60°C overnight. After hybridization, slides were washed in a wash solution [50% formamide, 2× standard sodium citrate (SSC)] at 60°C for 30 min, treated with 6.25 μg/ml RNase A (30142-04, Nacalai) in Tris-NaCl-EDTA buffer (10 mM Tris-HCl (pH7.6), 1 mM EDTA, 0.5 M NaCl) at 37°C for 30 min, and washed at 60°C in 2× SSC for 20 min and twice in 0.2× SSC for 20 min. Slides were then blocked with Tris-NaCl-Blocking (TNB) buffer [0.1 M Tris-HCl (pH 7.6), 0.15 M NaCl, 5 mg/ml blocking reagent (FP1020, PerkinElmer)] for 1 h at room temperature.

Signals were detected immunocytochemically by a combination of peroxidase (POD)-conjugated antibody and tyramide signal amplification (TSA) system. To obtain clear signals, we used the “enhanced POD-TSA reaction” protocol (28, 29), instead of the manufacturer's standard TSA protocol. After blocking, slides were incubated with anti-DIG-POD (11207733910, Roche) diluted in TNB buffer (1:500) one or two overnight at 4°C, washed three times in Tris-NaCl-Tween20 (TNT) buffer [0.1 M Tris-HCl (pH 7.6), 0.15 M NaCl, 0.05% Tween20] for 5 min, further rinsed three times in borate buffer [0.1 M H_3_BO_3_ (pH 8.5), 0.1% Tween20], and incubated with TSA reaction solution (20 mg/ml sodium dextran sulfate, 0.3 mg/ml 4-iodophenol, 0.003% H_2_O_2_ in borate buffer) containing TSA Plus Cy3 Reagent (1:200; TS-000202, Akoya Biosciences, MA, USA) for 30 min at room temperature without shaking. After final washing in TNT buffer, sections were counterstained using 4',6-diamidino- 2-phenylindole (DAPI) and mounted in Fluoromount/Plus (K048, Diagnostic BioSystems, CA, USA). Fluorescent images were captured using a TCS SPE confocal system (Leica Microsystems, Germany).

When applied to multicolor staining, after the first TSA reaction, slides were washed in 0.1% Tween20 containing PB (PBT) and incubated in 3% H_2_O_2_ containing PBT for 10 min at room temperature to inactivate POD, followed by three times washing in TNT buffer. Slides were then incubated with another POD conjugated antibody, and we performed the above-mentioned signal detection process repeatedly. FLU- and DNP-labeled riboprobes were detected in combination with 5-(and-6)-carboxyfluorescein tyramide (29) (kindly provided by Dr. Yasuko Akiyama-Oda) and TSA Plus Cy5 Reagent (TS-000203, Akoya Biosciences), respectively. In our protocol, the order of detection greatly affected the signal intensity (signals of the first detected genes were stronger than those detected later). In addition, DIG-labeled riboprobes tended to provide better signals than FLU- and DNP-labeled probes. Therefore, in multicolor detection, we used DIG-labeled probes for a gene whose expression level was lower than the other target genes.

## Results

### Identification and Phylogenetic Analysis of OBP Genes in *P. xuthus*

**Figure 1:**
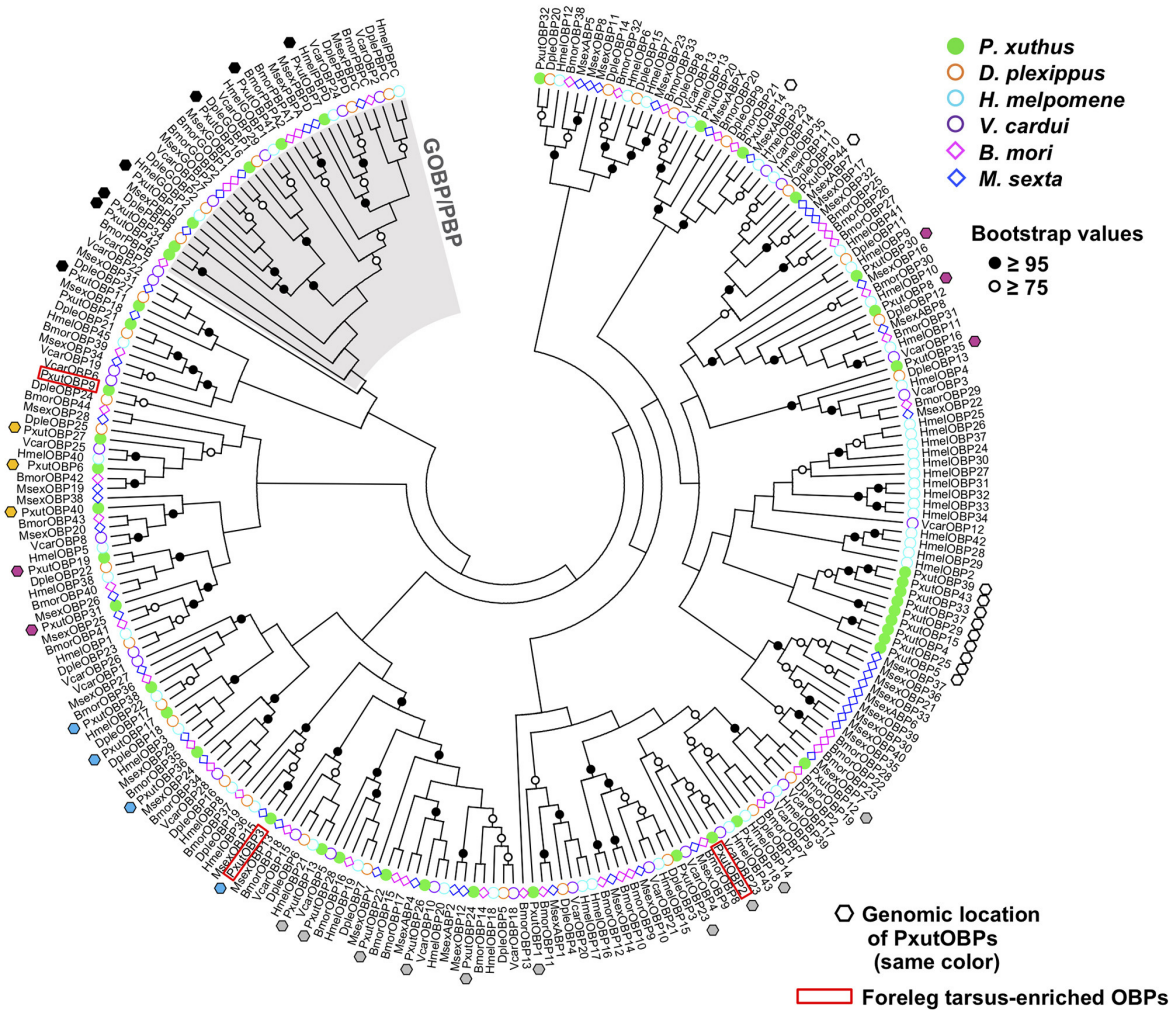
NJ tree of OBPs from *P. xuthus* and other lepidopteran species.

By searching the genomic database of *P. xuthus* and our RNA-sequencing assemblies, we found in total 44 OBP genes, including three previously reported genes (*PxutOBP1, 2*, and *3*). Most of the OBP genes were clustered on the genome; 38 genes resided on only six scaffolds, each of which had 4–11 OBP genes (Figure 1, Supplementary Data Sheet 2, and Supplementary Figure 1). Based on the cysteine patterns of translated proteins, 21, 16, and 7 genes were classified as the classic, minus-C, and plus-C OBPs, respectively (Supplementary Figure 1). The names of newly characterized 41 OBP genes were designated according to the order of expression levels in the foreleg tarsi (see below). Regarding the overall profile of OBPs, the NJ tree indicated that *P. xuthus* has GOBP/PBP complex, which is monophyletic OBP genes specific to Lepidoptera, and genes locating on the same scaffolds tend to be phylogenetically close, consistent with previously characterized lepidopteran species (25, 30) (Figure 1).

### Expression Profiles in Appendages

**Figure 2:**
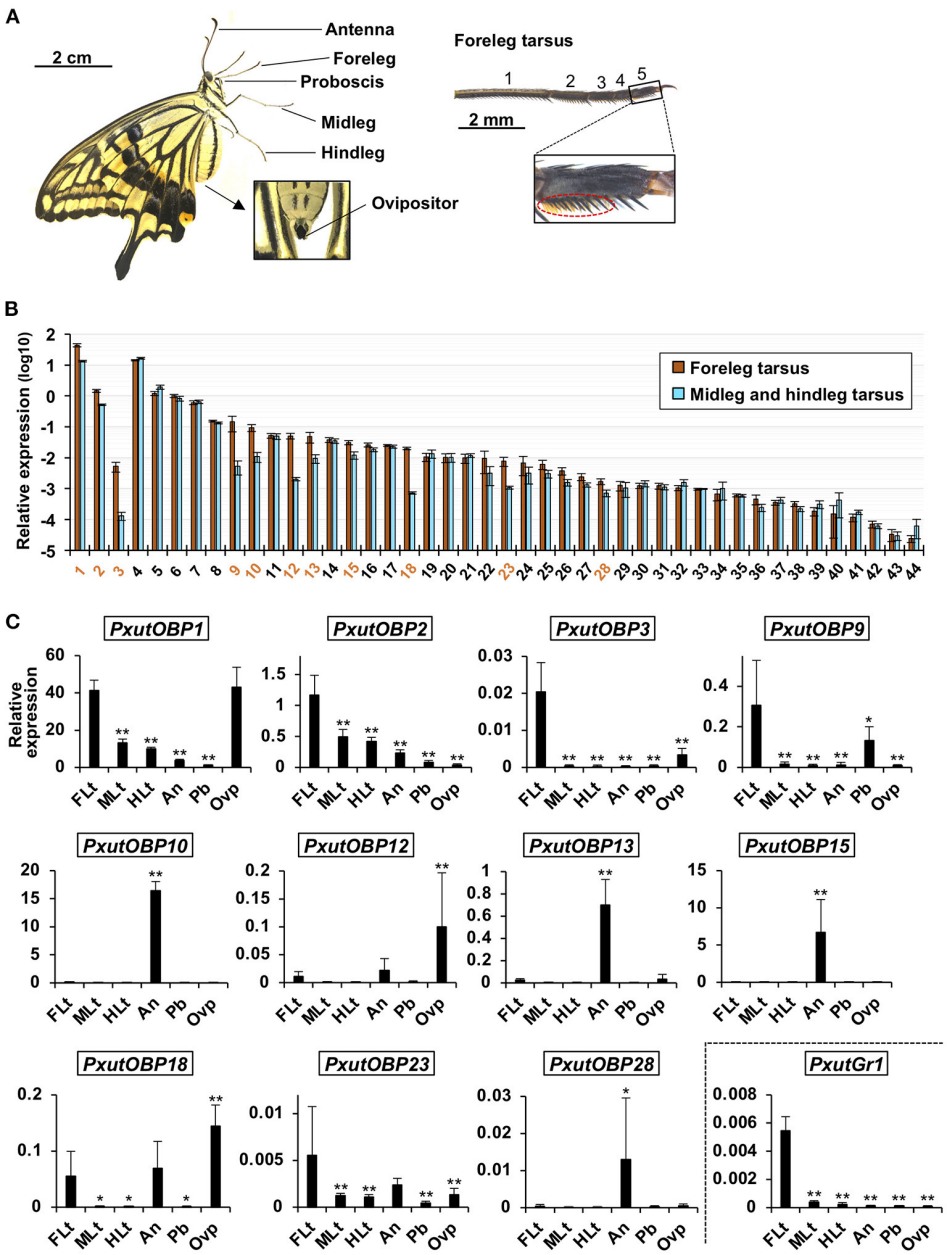
Expression levels of identified *PxutOBP*s. **(A)** Appendages examined for gene expression with a magnified view of the foreleg tarsus. Orange brushes in the dotted ellipse are the gustatory sensilla. As few gustatory sensilla are distributed in the first tarsomere (11), we collected the other four tarsomeres as “leg tarsus” samples. **(B)** Comparison of expression levels of *OBP*s between the tarsus of the forelegs and that of the other legs sampled from 0-day-old adult females performed by qRT-PCR. Three individuals were used per lot. All data are shown as the means ± SEM (*n* = 5, each leg). Student's *t*-test was conducted. OBP genes expressed significantly higher in foreleg tarsus (*p* < 0.05) or exhibiting prominent average fold change (the ratio of expression levels in foreleg tarsus to those in the other leg tarsus > 5) were indicated by orange-colored numbers. **(C)** Comparison of expression levels of *OBP*s among the foreleg tarsus (FLt), midleg tarsus (MLt), hindleg tarsus (HLt), antenna (An), proboscis (Pb), and ovipositor (Ovp) sampled from 0-day-old adult females performed by qRT-PCR. Two individuals were used per lot. All data are shown as the means ± SD (*n* = 6, each appendage). Multiple comparisons were performed using Dunnett's test. Asterisks indicate significant differences compared to expression levels of the foreleg tarsus (*, *p* < 0.05; **, *p* < 0.01).

We first compared the expression levels of 44 OBP genes between the tarsus of forelegs and that of other legs (Figure 2A) by qRT-PCR analysis. Eleven OBP genes (*PxutOBP1, 2, 3, 9, 10, 12, 13, 15, 18, 23*, and *28*) showed significantly higher expression levels or tended to be preferentially expressed (>five-fold) in the foreleg tarsus (Figure 2B). Most of the insect OBP genes are known to be expressed in the antennal olfactory sensilla (16, 17). We further examined expression levels of these 11 OBP genes among 6 appendages (foreleg tarsus, midleg tarsus, hindleg tarsus, antenna, proboscis, and ovipositor) (Figure 2A). qRT-PCR and statistical analyses revealed that *PxutOBP10, 13, 15*, and *28* were expressed predominantly in the antenna, and *PxutOBP12, 18*, and *23* were highly expressed in the antenna as well as the foreleg tarsus (*PxutOBP12* and *18* showed the highest expression levels in the ovipositor) (Figure 2C). Thus, we focused on the remainder (*PxutOBP1, 2, 3*, and *9*) in subsequent experiments.

### Visualization of *OBP* Expression in the Foreleg Tarsus

**Figure 3:**
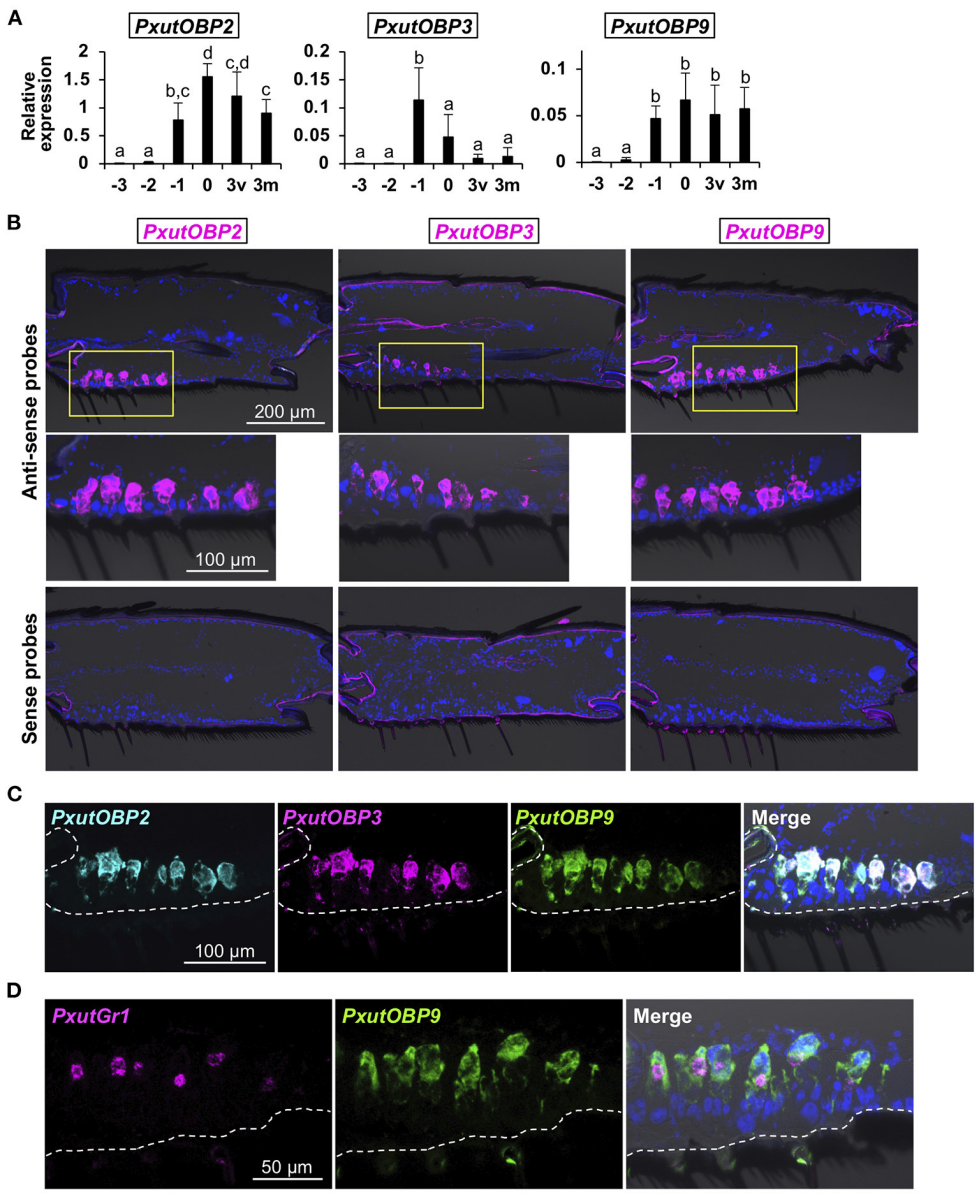
Visualization of *PxutOBP2, 3*, and *9* expression in the foreleg tarsus. **(A)** Developmental time-course of the expression of *PxutOBP2, 3*, and *9* in the female foreleg tarsus analyzed by qRT-PCR. −3, −2, −1: 3, 2, and 1 day before eclosion, respectively; 0: the day of eclosion; 3v: 3-day-old virgin female; 3m: 3-day-old mated female. Two individuals were used per lot. All data are shown as the means ± SD (*n* = 5, each developmental stage). Multiple comparisons were performed using Tukey-Kramer's test. Different letters indicate significant differences (*p* < 0.05). **(B)** Spatial distribution of *OBP*-expressing cells in the female fifth tarsomere visualized by FISH. As shown in (**A**), *PxutOBP3* expression was peaked on 1 day before eclosion. Therefore, −1-day-old females were used for detection of *PxutOBP3* transcripts while 0-day-old females were analyzed for the visualization of *PxutOBP2* and *9* expression. **(C)** Simultaneous detection of *PxutOBP2, 3*, and *9* expression in the female fifth tarsomere by triple-color FISH. −1-day-old females were used. DNP-, DIG-, and FLU-labeled riboprobes were synthesized for *PxutOBP2, 3*, and *9*, respectively. **(D)** Simultaneous detection of *PxutGr1* and *PxutOBP9* expression in the female fifth tarsomere. 0-day-old females were analyzed. DIG- and FLU-labeled riboprobes were synthesized for *PxutGr1* and *PxutOBP9*, respectively.

Under our laboratory conditions (25°C, 16L8D), it took 9 days for *P. xuthus* to complete pupal metamorphosis. Adult-like legs could be observed 3 days before eclosion (−3 days). We sampled foreleg tarsi of −3-, −2-, −1-, 0-, and 3-day-old females and quantified the expression levels of *PxutOBP2, 3*, and *9*. Significant upregulation of OBP genes was detected at −1 day, and transcription continued after adult eclosion, although *PxutOBP3* expression was decreased at relatively low levels (Figure 3A and Supplementary Figure 2).

Next, to examine spatial expression patterns, we performed fluorescence *in situ* hybridization (FISH) analyses with fresh-frozen sections. Since the gustatory sensilla are most abundant in the fifth tarsomere (11, 31), subsequent analyses were performed using the fifth tarsomeres of female forelegs. *PxutOBP2, 3*, and *9* were expressed at the bases of the gustatory sensilla (Figure 3B). In contrast, *PxutOBP1* signals were broadly detected in the tissues along the ventral cuticle (Supplementary Figure 2), suggesting non-chemosensory function of this gene, and thus we excluded *PxutOBP1* from the subject of further FISH analysis.

Triple-color staining successfully detected co-localized signals of *PxutOBP2, 3*, and *9* (Figure 3C). Because the amounts of transcripts of insect gustatory receptor (Gr) genes are generally quite low (32, 33) (Figure 2C), visualization of *Gr*s expression by *in situ* hybridization has been thought to be difficult (34). Preliminary experiments showed that our FISH protocol (see section Materials and Methods) was effective to visualize the expression of a previously identified synephrine (one of the oviposition stimulants) receptor gene *PxutGr1* (12) (Supplementary Figure 3). Then, we next performed simultaneous detection of *PxutGr1* and *PxutOBP9*. *PxutGr1*-expressing small cells (gustatory receptor neurons) were observed in close proximity to *PxutOBP9*-expressing large support cells (Figure 3D and Supplementary Figure 3). These results indicated the coexpression of all the foreleg tarsus-enriched OBP genes in the oviposition stimulants-responsive sensilla. Similar localization patterns were also observed in males (Supplementary Figure 4). This is in agreement with a previous finding of no sexual dimorphism in the electrophysiological response of foreleg gustatory sensilla to the oviposition stimulants (11), although ecological significance has been unknown. Lower expression levels in males than in females (Supplementary Figure 4) possibly resulted from a smaller number of gustatory sensilla on the foreleg tarsus of males (11, 31).

### *OBP* Expression in Other Appendages

**Figure 4:**
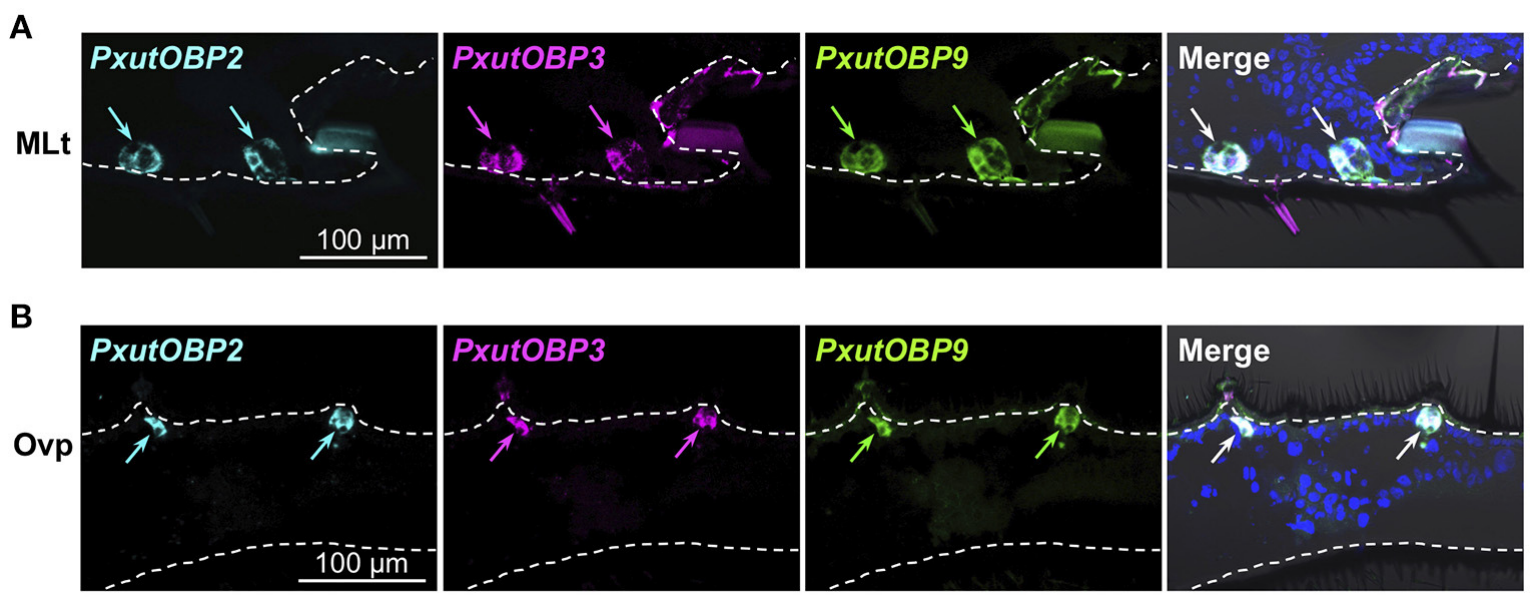
Coexpression of *PxutOBP2, 3*, and *9* in the midleg tarsus **(A)** and ovipositor (papilla analis) **(B)**. −1-day-old females were analyzed. DNP-, DIG-, and FLU-labeled riboprobes were synthesized for *PxutOBP2, 3*, and *9*, respectively. Arrows indicate signals of *OBP*s expression.

**Figure 5:**
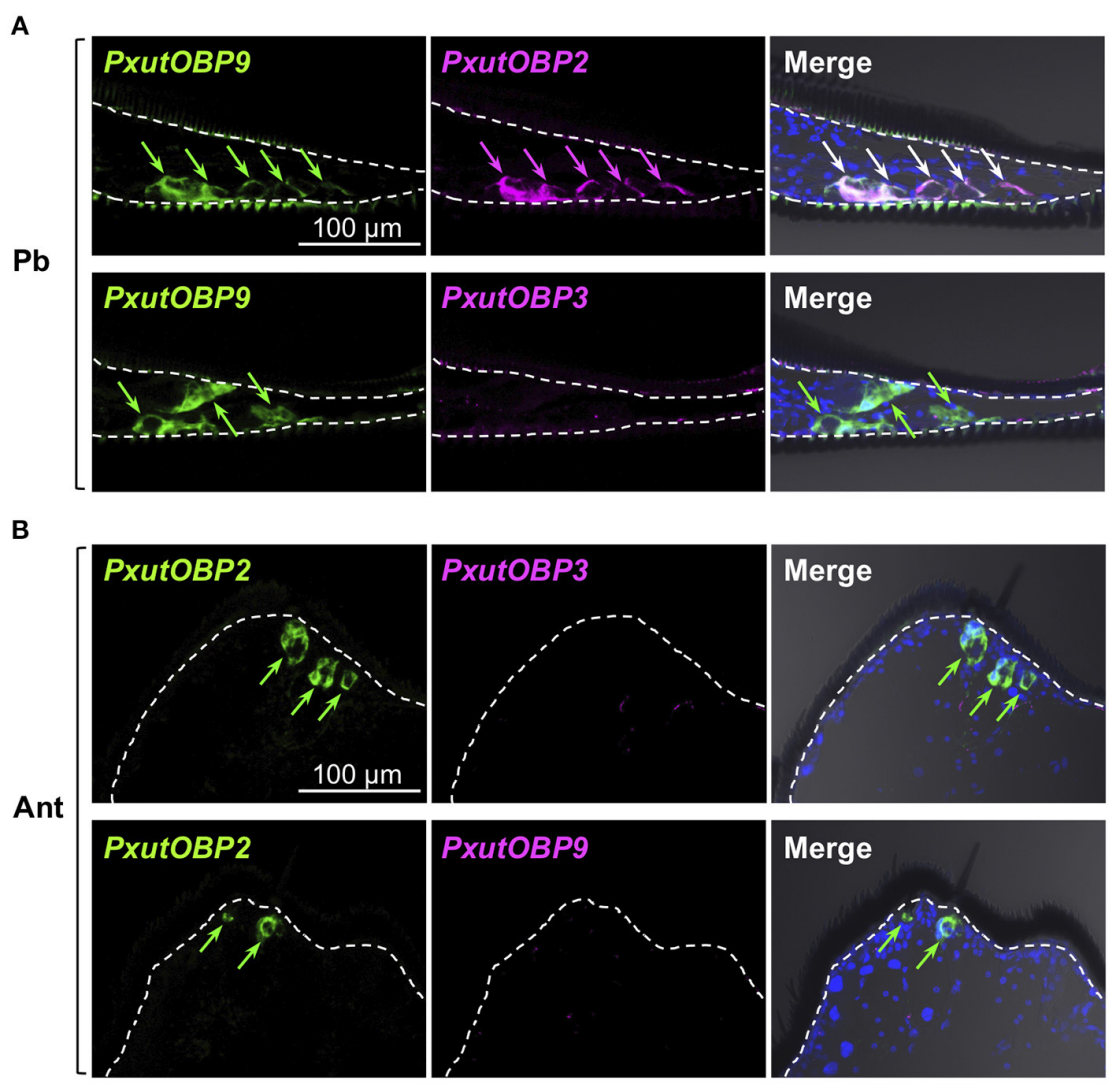
Visualization of *PxutOBP2, 3*, and *9* expression in the proboscis and antenna. Simultaneous detection of two different pairs of OBP genes was performed. −1-day-old females were analyzed. Arrows indicate signals of *OBP*s expression. **(A)** Longitudinal sections of the proboscis. FLU-labeled riboprobe was synthesized for *PxutOBP9*. For detection of *PxutOBP2* or *3* transcripts, DIG-labeled riboprobes were used. **(B)** Longitudinal sections of the tip of antenna. FLU-labeled riboprobe was synthesized for *PxutOBP2*. For detection of *PxutOBP3* or *9* transcripts, DIG-labeled riboprobes were used.

Insects can taste chemicals by various body parts (35, 36). FISH analyses revealed that *PxutOBP2, 3*, and *9* were also coexpressed in the other legs and ovipositor (Figure 4 and Supplementary Figure 5). On the other hand, no *PxutOBP3* signal was detected in the proboscis, whereas co-localized signals of *PxutOBP2* and *9* were again observed (Figure 5A). We only found *PxutOBP2*-expressing cells in the antenna without any signals of *PxutOBP3* and *9* (Figure 5B).

### *OBP* Expression in Larval Gustatory Organs

**Figure 6:**
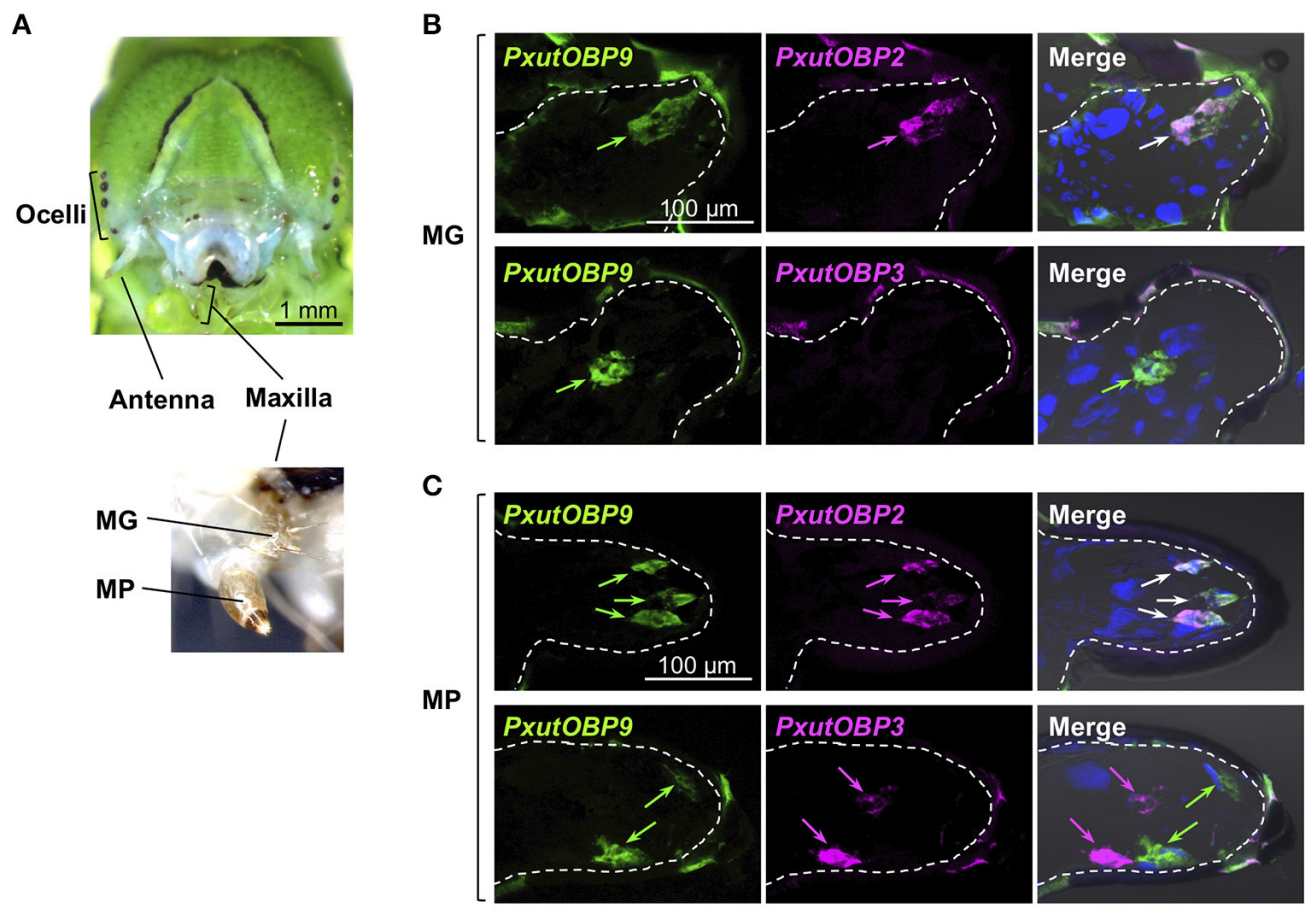
Visualization of *PxutOBP2, 3*, and *9* expression in the larval gustatory system. **(A)** Frontal view of a head of a final instar larva with a magnified view of the larval maxilla. **(B,C)** Simultaneous detection of two different pairs of OBP genes in the maxillary galea (MG) **(B)** and the maxillary palp (MP) **(C)**. FLU-labeled riboprobe was synthesized for *PxutOBP9*. For detection of *PxutOBP2* or *3* transcripts, DIG-labeled riboprobes were used. Arrows indicate signals of *OBP*s expression.

Unlike adults, the vast majority of lepidopteran larvae feed on plant tissues (4, 37). The major gustatory organ of larvae, the maxilla, mainly consists of two parts called the maxillary galea (MG) and the maxillary palp (MP) (36) (Figure 6A), and chemosensory neurons housed in MG and MP contribute to food choice (38, 39). We investigated the expression patterns of *PxutOBP2, 3*, and *9* in the MG and MP of *P. xuthus* fifth instar larvae. In the MG, similar to the adult proboscis, co-localized signals of *PxutOBP2* and *9* were observed, whereas *PxutOBP3* signal was undetected (Figure 6B). In the MP, *PxutOBP3* with *PxutOBP2* and *9* signals were observed, although *PxutOBP3*-expressing cells differed from *PxutOBP2* and *9* coexpressing cells (Figure 6C).

## Discussion

In the present study, we performed genome-wide searching and found 41 OBP genes (Figure 1), besides the previously identified *PxutOBP1, 2*, and *3* from EST analyses of female foreleg tarsi (24). Vogt et al. summarized the OBP genes found in the four lepidopteran genomes (*D. plexippus, H. melpomene, M. sexta*, and *B. mori*) (25). The numbers of OBP genes in these species are ~30–50, similar to that of our identified *PxutOBP*s. Three OBP genes, *PxutOBP2, 3*, and *9* showed enriched expression in the foreleg tarsus (Figure 2). By applying multicolor FISH protocols, we demonstrated the coexpression of these three OBP genes at the bases of the gustatory sensilla of the foreleg tarsus (Figure 3).

Among the adult appendages which we investigated (Table 1), *PxutOBP2* and *9* were commonly coexpressed in the same cells, except for the antenna where only *PxutOBP2* signals were detected (Figures 4, 5). In the proboscis, *PxutOBP3* signal was never observed (Figure 5). By contrast, besides the foreleg tarsus, co-localized signals of *PxutOBP2, 3*, and *9* were also observed in other legs and the ovipositor, both of which could have direct contact with the leaf surface during the drumming and egg-laying behavior. A small but considerable amount of *PxutGr1* transcript was detected in the midleg and hindleg by our qRT-PCR analysis (Figure 2C). These legs may serve an ancillary role in host plant recognition. Some insects employ gustatory information received by the ovipositor to evaluate the oviposition substrate (40, 41). Not only photoreception and mechanosensation (42) but taste perception on the ovipositor could affect the final decision on egg deposition in *P. xuthus*. *PxutOBP2, 3*, and *9* were also expressed in the larval MP, whereas no *PxutOBP3* signal was observed in the MG (Figure 6 and Table 1). Since the proboscis in adults is developmentally derived from the MG in larvae, the similarity in the undetectable expression of *PxutOBP3* is interesting. Recently, in *B. mori*, Tsuneto and colleagues reported that gustatory neurons housed in MP were tuned into feeding stimulants derived from mulberry leaves (host plant of *B. mori*), whereas those in MG responded to sucrose (39). Based on these findings, they proposed fascinating model; the MP contributes to host selection *via* the detection of unique combinations of phytochemicals, and the MG participates in nutritional evaluation (39). Taken together, it is plausible that *PxutOBP2* and *9* are basic OBPs of gustatory systems, and cooperation with PxutOBP3 enables *P. xuthus* larvae and adults to detect the cue phytochemicals leading to make appropriate decisions.

**Table 1:** Topographic expression patterns of *PxutOBP2, 3*, and *9* among the appendages based on the results of FISH experiments.

	**Adult**					**Larva**	
	**FL**	**ML, HL**	**An**	**Pb**	**Ovp**	**MG**	**MP**
*PxutOBP2*	+	+	+	+	+	+	+
*PxutOBP3*	+	+			+		+
*PxutOBP9*	+	+		+	+	+	+

In the case of *P. xuthus*, 10 and 11 compounds have been identified as oviposition stimulants (10) and larval feeding stimulants (43), respectively. Interestingly, the components are quite different in the two sets of stimulants, and only stachydrine is commonly included, raising the possibility that PxutOBP3 interacts with stachydrine. It is also possible that PxutOBP3 is involved in avoiding the unpreferable plant species. Hydroxybenzoic acid derivatives have been known as deterrents to both oviposition and larval feeding (44). These compounds are contained in *Orixa japonica*, which is rutaceous plants but always rejected by *P. xuthus*. Alternatively, because insect OBPs show highly diverse binding affinity and selectivity [i.e., some OBPs bind to specific ligands, but others interact with a variety of compounds (15, 17, 45), it is not surprising that PxutOBP3 would interact with a broader spectrum of phytochemicals. To identify the ligand for PxutOBP3, future biochemical study is required. Intriguingly, a previous study showed that OBPs which coexpressed within the same olfactory sensilla in the antennae of *Anopheles* mosquitoes can form heterodimers with novel ligand specificities leading to amplify the number of volatiles that can be perceived (46). To uncover the role of PxutOBP3, future identification of the ligand by biochemical analysis and loss of function experiment with careful consideration of the cooperative interactions between coexpressing-PxutOBP2 or 9 is particularly important.

Phylogenetic analysis indicated that OBP genes orthologous to the foreleg tarsus-enriched *PxutOBP2, 3*, and *9* were conserved in other lepidopteran species (Figure 1). Comparative analyses of these orthologous genes, particularly *PxutOBP3* orthologs, across a broad range of species would provide insight into the functional significance of OBPs in host plant selection in Lepidoptera. Furthermore, although we focused on OBP genes preferentially expressed in the foreleg tarsus in this study, longer-distance olfactory information also plays an important role in the process of host plant recognition (5, 7, 47). Therefore, the *OBP*s highly expressed in the antenna are important targets for future analysis.

Insects utilize diverse chemical signals for survival, and thus, the study of the chemosensory-related molecules is a fundamental topic in the field of insect science. Recent progress in the next-generation sequencing techniques leads to the comprehensive identification of chemosensory genes in various insects, including “non-model species” (17, 27, 48–52). Because chemosensory organs have many sensilla housing multiple sensory neurons each of which responds to different stimuli in general (35, 53, 54), investigation of cellular-level expression patterns of chemosensory genes is fundamental for understanding the molecular and neural mechanisms of chemosensation. In the non-model insects, however, spatial distribution pattern of chemosensory-related molecules has not been well characterized (46, 48, 49). The multicolor FISH analysis applied in this study allowed us to clearly visualize the low-level transcripts of *Gr1* and the coexpression of three OBP genes in *P. xuthus* (Figures 3, 4). We expect that our protocol is an effective approach to gain spatial and combinatorial expression patterns of the chemosensory genes in other non-model insects.

## Data Availability Statement

The datasets presented in this study can be found in online repositories. The names of the repository/repositories and accession number(s) can be found below: DDBJ [accession: PRJDB11535, PRJDB11545].

## Author Contributions

AU and KO conceived and designed the study and performed the bioinformatic analyses. AU carried out the molecular lab work, analyzed the data, and wrote the manuscript. All authors gave final approval for publication.

## Conflict of Interest

The authors declare that the research was conducted in the absence of any commercial or financial relationships that could be construed as a potential conflict of interest.

## Publisher's Note

All claims expressed in this article are solely those of the authors and do not necessarily represent those of their affiliated organizations, or those of the publisher, the editors and the reviewers. Any product that may be evaluated in this article, or claim that may be made by its manufacturer, is not guaranteed or endorsed by the publisher.
