# Supplementary material for: Coexpression of Three Odorant-Binding Protein Genes in the Foreleg Gustatory Sensilla of Swallowtail Butterfly Visualized by Multicolor FISH Analysis
Source: Front Insect Sci. 2021 Jul 30;1:696179. doi: 10.3389/finsc.2021.696179 (PMC10926539; doi:10.3389/finsc.2021.696179)
Supplement: Supplementary file 1 [file Data_Sheet_1.PDF]

Supplementary Material

Supplementary Data S1

Amino acid sequences of OBPs in other lepidopteran species used for phylogenetic tree construction. For OBPs of *D. plexippus*, *H. melpomene*, *M. sexta* and *B. mori*, sequences listed in Vogt et al. 2015 were used. OBP sequences of *V. cardui* was kindly provided by Mr. Hiromu C. Suzuki.

>DpleGOBP1

MTSRSGRRLLLLLLVQLAVQLKANVDVMKDVTLGFGEALKLCREESQLTEEKMEEFFHFW
RDDFKFDDRAVGCAIKCMSSHFDLLTDSHRMHHRNMDNFIKSFPNGEVLSQQMVTLIHECE
QQHDSEEDHCWRILRVAECFKSSCKKHGIAPTMELLMAEFVMESEAN

>DpleGOBP2

MAVRGLLLLSAVLAATSVTVDGTAEVMSHVTAHYGKSLEECRKETGLSKEILEEFKHFWSD
DFEIVHRPFGCTLICMSNKFALLQDDARMHHINMNDYVKGFPEGDVLADKLVQMIHKCEKE
YDDIKDDCDRVVKVAACFRADAKKEGIAPEVAMIEAVMEQY

>DplePBPB

MAIMMFAIFILSIFIPSVNLNQDVMKSLSYKFGTKLFECGERTNYTRAMARDILHIWEESYDL
NHDETGCLVLCAMVRLELLDQQGNMIVENTEGFIRANGGDDSMVSFLIQLYSMCREKTSSIS
NGCKAAIELSKCFRAAIQQIGWVPDTTSLLVISYD

>DplePBPC

MNKSCVFVFALIFVNVQKVQSNEVMKGITSSFFKVLDECKRELGLTDNVLTDLYYFWKQDH
PLMHRDTGCAIVCMSQKLNLLDTIGKLHHGNAQEFAINHGAGEQMAKKLVTMVHECEQQF
MEQEDSCLRALDVAKCFRTAMHDVNWAPKFDIIVTEVLTEVK

>DplePBPD

MALFCWRLLFAAGVLSLAQGTLASQEIMKKLTTGFVKAMEECKAELNLGDHIIQDFMNYW
REEYELLNRDTGCAIMCMASKHDLITEDMKIHHENAHEFAKSHGADDDLAKQLVQMIHDC
EKQFTDITDDCSKTLEISKCFRTKIHELKWAPSMETILEELMTET

>DpleOBP2

MKKWFLELTVECSKEHPVTKEEIQMLKDHKIPDNKNVKCLMGCVFRKIGWLDDNGMFSFN
NAYKTSEEEYPDDKTKLEKAKNLYSLCEKVNTAEVSDGKEGCERSSLLAKCLIENSSKMGF
VVQ

>DpleOBP3

MTAEQKQMVHQHFEQVGMECIKTHEITAEDVTNLRTRKIPTGENASCFLSCIFKHVGIMDDN
GLLQKESAIELAKKVFDDEEELKLIEDYLHSCSSVNTATVSDGEKGCERSLLAYKCMIENAS
QFGIDL

>DpleOBP4

MMSLKYLFVFGVLAVSLRSARAISDEDRNKIHTAVLPHVAECSANFGVTEDDIKAAKEAGT
LGTFNPCLMGCVLKKIQVIDDKGLFDADKAVELSNKYFSDEADKKKAEEIITTCKAVNDKE
VGDGEKGCERAGLLFQCFLPFKDAFIN

>DpleOBP5

MTRQQIKNSSKMLKKNCMGKNDVTEDMVGEIDKGKFIEDKNVMCYIACIYQMSQLVKNNK
LNYEASIKQVDLMFPPDMKEAMKASIENCKDISKKYKDICEASYWTAKCIYDDNPKNFIFA

>DpleOBP6

MYILAVVKFLAALTICEAMTMKQIKSTGKMMRKTCQPKNNVADDKIDDIGTGVFIEEREVM
CYVACIMKMANAIKNNKLNYEAAIKQADLLLPDEIKEPTKEAITACRKVADSYKDICEASFH
VTKCIYNHNPSIFFFP

>DpleOBP7

MTRAQLKKTMTIMKNQCMPKHNVNNDKVGQIEQGVFIEDHDVMCYIACIYKTIQVVKNNR
LDKDLISKQIDALYPPELKESTKEAVSKCIKEQAKYEDPCAGIFYACKCLYEDNPANFIFP

>DpleOBP8

MAELAKMLRDNCLEETGADVALIDKVNQGATLMPDGKLKCYIKCVMETAGMMSGGEVDV
EAVVAVLPEELQRHADTMRACGTKRGLDDCDTAFLTQACWQAGCPKDYILI

>DpleOBP14

MNTWSILFTLTIILLGTINCYKHSKYLTKSLDSEPSLSILYARDKKSDTITNECLMEMYPRNLY
KYPLHIDRNDIPCIIHCVLKKFGIMSNDGIINIRNYYRRVQAIHRYDPRVLISDVGETCAQNIN
GMNLDHDVCKKAKVFNDCTQLYVISYKDLED

>DpleOBP15

MYAHEKISDMVAQQCLTEMYPKGKKIELQESDESCIIYCVLKKFGIINGNGQFNLDIYRKRV
QMAHQLDSRNLMNDKGGACVESAEATQHKQDVCKKAKVFNDCTHLYRMIF

>DpleOBP16

MQLYVFLCLIGHVLASKSHGYSGTLVDFTDPKVQGHLDALVRMAQSCVIKVRASPKDVRA
YFTNSPPITRSGQCFAACMMEQSDVINHGKVNRELLIHLASLVNGKNSGVVRKLHSISRLCL
DSIEGMSDRCQLASTYNDCLNENMIEFAFPLDIAEEAVRKMPFHLIQPK

>DpleOBP17

MTKEEQIDRFNKMNEEVEPFRQNLTDCARQVKASMIDVEHFLKRIPQSSMQGKCFVACILKR
NSIIVNNKISRENLLEANRAVYGQDSEVMTRLNTAIVECSDVVEGIFEVCEYSSVFNDCMHM
KMEHILDKVTMERRMEALGQMTSDPDVWTDEDDEILKLIKDEL

>DpleOBP18

MFLRFLFPLMFFVYVETDQAVENNRMVGVDTVHDIKIDKDTIITRNMNLKKKNEKSSEAQIE
QKNVSPDWSYSSFPEDVKKHVDQFKRNMSECLKEVHASDKRPVKRLSPKKESPVHGDCLIA
CVLKRNSVIENGKIHKENLIALVKKFYEKDEKLMKKLERNLDRCIETSARDKDDCAIAARLN
ECTNDIMTSNKHKIVVNY

>DpleOBP19

MNLYGVHPYVKLSPERFYLWTDSPIYAVVHKTLSMTARSCMVSANASEVDLEPLREDPPFP
EKSACILNCLLEKINIVKSGKFSKSGFMTTISPLVFTNKKKMDHMKTVSENCDKEVNHDVSS
CELANEITSCIYKYAPELHFKN

>DpleOBP20

MRVLVVAAVTLITITLTRCVPRTDKMQNNVKDLTSTTPNSMEEQMDKISNDLRPVMAECNE
TFRIEMYYLETLNTTGSFPEELDRTPKCFLRCVLEKAEVASADSQFDVSRTADVFDQIRVLPH
DDLVKMATTCSDRAETCKCERAYQYLKCLMGMIINKYDTT

>DpleOBP1

MECSKDYPLTDEDIAQLKDKQFPDKDDVRCLFACAYKKTGMMDDQGKLSVDGVNNLAKK
YFSDDQDKLQKSQKFTEACAGVNDEAVTDGEKGCERAALIYKCSIEQASQGLTDEEVKKEFI
KEVMTCTKDITVDMFDLMELEQLKVPTKTNVKCVLACAYKRVGTMNKEGKYDIKEAYKIS
ETMMKGDDKRIENGKKLADLCSKVNEADVRDGNKGCERAALLFKCVIENAPKLGFKV

>DpleOBP9

MPSLSLLLYLAMLTLCYGAKEKPELSEEIKEIIQHVHNECVGKTGVAEEDIANCENGIFKEDM
KLKCYMFCLLEEGSLVDDDGNVDYDMMVSLIPDQYTDRVLNMINGCKHLVLSISCDDSETS
GMQVPLEAPKPSSSPSAPHPRRGLDSDIGPVENTPRIFA

>DpleOBP10

MKFLVLICVCAIFISRSNAQNAHLTSAQKEKVKQYTAECIRESGVKHEVLADAKKGNLNEDE
GLKRFIFCFFQKSGIVTADAKLNMEVALSKLPKDIDKVAAGKVLSECKNKNGKNHADTAFQI
FKCYHKATKQHVLFE

>DpleOBP11

MSTKFYCILAIVLFFFVQVRNTASITNYKSPLIAEYVEHCLRESRANPEHIIFLKNGVIHSPNYA
LKNWLLCYLSRTGVMSPEGVLKQHVVMKKVAKQDKDLVEKIIDKCLFKTPHEPVDTAWKY
LTCFRKRQPQYAREINHI

>DpleOBP12

MKTFIVLAAILVAAQALTDEQKQKLNKHKSECLAEFKPDEQLVEKLKAGDFKTENEPLKKY
VLCMLMKSELMTKEGKFKKDVALAKVPNPEDKPAVEKLIDACLANKGNTPQQTAWNYAK
CYHEKDPNHSIFQ

>DpleOBP13

MCYWISTTGRDSEDDVVRILPSDVYKTNDEYRHIWSPVHGDAVVFVRPLLFHRLVVVLLQV
SLQLSQDDMLTLLVVLLLHQVTTESKSFMPLGKAERAMFLSHSEACLEQSGAERAQVERLV
GGAPEDSPALRRHVLCVLRRCKLLRKDGRLDKHALRDRISASNDTKILEGCSDQSGDTPEDL
AWHLFRCGLNKKVLFEHMTAAAPNEA

>DpleOBP27

MEEEISNALQDCSQLNDKLNIKVNIEKRQRRHNDFRYYAFRIDANSKQEINQYNHERRNTST
ENDKNINGNDSGINFSRYPYGGIEEDLINSGNRQTIDDDRKSNLKQQSRFKRNEPLVSKDDVD
KCLSQCVFANLQVVDSKGIPREAMLWNKIQSSVTSEQSETLMREQIRSCFQELQSESEDNGC
VYSNRLERCLMLHISDRNRNSTQTYTTSIK

>DpleOBP21

MVSKISVLLCCIYVFGISLSDSAISADSESRCRNPPTAPQKIERVITLCQDEIKLSILREALDVIK
EEHTMPAQRRRNKREVPFTHDEKRIAGCLLQCVYRKVKAVDGYGFPTLEGLVGLYSDGVN
ERGYFMAVLEASRECLMKNHDLFSRTVPMDNGRNCDVSFNIFECISDRIGEYCGNSGL

>DpleOBP22

QAGFGRSYMGNSRLCREWSCFNKKLNLTDSLPPKEHYGLILKNRFPESWQTVVDSALEVCY
GKRSRKYTNTCPGQAIMFCVMDQAVMNCPINSLTTDSCTPLSSLAGYNYMFSQSRYENLEQ
NLKLDRRPPAFLRNVSYYYHTTCCDVPDIFEEDMLKECGFERFVHYNVHGVQQKEANIEFH
TTSPITSTSGISNEFIHDENLNEVAADESEDPLDCCDVSGFIEPSWKSECGFQLKWDVKNRLTI
LNSDVPTSTASTLRDMDIKVVPITCENQQCIFDRLGITKSGEVDVDKFVKLLDNMTNREPLW
NRAKERVLASCLRKPLIGYESDCEINSILGCTLDVLSENCPHETREDQCKNSTNSKEGVICHIS
SSKYGPKNRRQFCNIPNLVRRDILDACGVSSIFKIEYVLPQSVDHSWGPGVNCKESTVSNCL
MNKMGVLNKYGFIDYFKMKDKMRSYSERLAASIYDLYTSSFINTPYYKDHCSSPKKLLNVI
DSILLTCPTQNRKKTKKCDKIFTEMKNMITDKIARDKIDEILNHNKNYYLPTNYIAKDSAQKT
KLTPLYDFGILSSNNIPPVKVIDVTPKFLLYPVHTTNNSSTLFSLHNDGVFRG

>DpleOBP23

MMKYLFVLICLSIATCEADDIASPVLCGVTPNAIYQCLGTPKVVKKEASSQCDSDLGECEKM
SCIFSKSGWMKDNKVDKEEFAAHFDQFAKENPDWKVAVEHMKSNCLSSDLPPQGVHLNCP
AYDVMICAFANFIKGAPASQWSSSSHCEYPRRFAASCPVCPTACFASAIPIGSCNACLSLPRSP

>DpleOBP24

MAEMTHLLTLFVLFLMSTVSTADEVDESCFELFDPEEPEQECCETDFETDDEVEYEEDFSDCP
TDFSTDEGKCDTIKCYYKHDGVWKDDDIDDEAVKTKLQSIDSKNPPAQRAAERILKHCLNG
KYMKYGTDDACPAMKYFLCSYINTVVECDSWNKTETCAKHSEYASKCKVSLG

>DpleOBP25

MLNLLRIALFIVAVNADLLNHERTKGATLKPISVCCDIPELGDPKPLAECSNMKLQGPCSDV
QCVFEKSGFLLDKQTLNKEGYRNHLMKWLEGHKEWKDGIEKAISDCVDVDLRQYLDYPCK
AYDVFTCTGIAMLKVVLSDVPPHCRGPPPGGANPSDCCKLPKVFTEEDFKECGIEKPTKERR
VPECSKQICLLKRYELMKDDTNVDKDAVAAFLDKYSEGDEDTQSAVEATKQKCLNNDLPNI
PEICEPSKIVFCVAGSMLMNCPKWDESEDCQKLKDHFENCNKYFPKK

>HmelGOBP1

MISLLTLSLACGVLTGLDATMEVMKDVTLGFGEALQSCREESQLTEDKMEEFFHFWRDDFK
FEDREVGCAMKCMSSHFNLLTDSHRMHHENTDKFIKSFPNGEVLSKRMISIIHTCEQQFDAL
EDHCWRILRIAECFKVACKKEGIAPTMELLMAEFIMEADPS

>HmelGOBP2

MAVRCYVVVFLALLALATLPRVRSTAEVMSHVTAHFGKSLEECREESGLSSDILDEFQHFWS
EEFQVVHRELGCAIICMSNKFLLMHDDARMHHVNMHDYIKSFPQGELLSEKMVNLIHNCEK
QFDDIEDECSRVVKVAACFKESAKEEGIAPEVAMIEAVLEKY

>HmelPBPC

MFKRLTVVVLVFVGLNKVTSNETLRGITASFLKVLEECRQELNIGDNVLADMYYFWKLDRT
LIHRDTGCAIVCMSKKLNLLDTSGKLHHGNAEEFALQHGAADDMAKKLVTTVHECEQKHE
LEEDQCLRALEIAKCFRGAMHEINWAPKVDVAISEILTEV

>HmelPBPD

MAVQKWQLILVMCVLMQACKVSPSQEIMHKLTKGFATAFEQCKQELNLGDNIMQDFLNY
WREEYELLNRDTGCAIMCMAQKHDLLTEDGIHHEKVHGFTKSHGADDELAKQLVTMIHEC
EKSNAGVSDECMKTLEVAKCFRTKIHELKWAPDMETILEEIMTDI

>HmelOBP3

MVGLKALHDIQINKDTIITRNMNLKTDNKISHNHDPDWSYSSFPKEVKSHVEQFKRNMSECL
KEVQLNDKRQVRRLSPKKESPVHGECLIACVLKRNGVIENGKIYKDNLLSLVRKFYGKDEK
LMKKLEKNVDRCIEASVKNKDDCTVASYLNECTNDLMANNKHKIIVNY

>HmelOBP6

MENISYKGKLFLLLFIAFAHVTLVDSYSHKFFSQNLDTEPSLSIQYARDKKSDMITNECLTEM
YPKNIYRYPLHIDRNDVPCIIHCVLKKFGIMSNDGVINTRNYYRRVRAIHRYDPRVLISDVGE
TCAQNINGMNLDHDVCKKAKVFNDCTQLYAISYRESDD

>HmelOBP7

MYAHEKMSDIVAEQCLNEMYPKGRRIQFEESDEPCIIYCVLKKLGIMNSNGQINVDM

>HmelOBP8

MLIHAVLALIGYTLAAQHRSFSGTMVDLSDPKVQGHLDALVRMAQSCVIKVRASPKDVRA
YFTNSPPITRSGQCFAACMLEQSDVINHGKVNRELLVHLAGLVNGKNSRVVRKLYGISRLCL
DSIEGMSDRCQLASTYNDCLNENMIEFAFPLDIAEEAVRKMPFHLIQPK

>HmelOBP12

MKIIIIITLALLTFSVAQCGSSVGNRMSTDDITTTTMATTMEDDDARSNFDVMSVMVDCNDT
FRVEMSYLESLNKSGSFPDETDKTPKCFVRCVLEKSDIVSGDSQFNVTRTAEVFSQIRDTSQN
DIIKMATACSDRPEKCKCERSYQYLKCLLETTIEINEMKSSK

>HmelOBP13

MQGFIVHCSIVAILVVGASALEGEMAELAKMLRDSCIEETGADIALIDKVNDGADLMPDPKL
KCYIKCVMETAGMMSQGAVDVEAVVAVLPPELQRHADKLRACGTKAGANDCDTAFLTQA
CWQGGCKEDYILI

>HmelOBP14

MKTLKLLILLFVFFFGDSQGMTDDEMREEFMRITMICSKDYKVDMKDLLSLQQLNIPTKKD
VKCLLACAYKKTGSMNKEGLYDIEASYRIAEMTKNGDPKRLENAKKLVDICAKVNDETVS
DGEAGCDRAGLIFKCVVENAPKVKNN

>HmelOBP15

AITDEQKAMIHSHFEMLGKECIKDNLISADDIKNLRAKKIPSGENAPCFLACMFKKLGIMDD
AGLLQKETVLDLARKVFNDEDEIKLIGDYLHSCSHINTESVGDGDKGCDRSMMAYKCMIEN
ASQVLFTFN

>HmelOBP16

MFLIVLIFQGYSDEERTKIYATMLPHILQCSTEYGITEDELKASKENEKFGSINPCFMGCIFKKI
HVINKEGIFNVEKAEKLSENFLVHDEDKKKASAVIKACATINDEDVSDGEKGCDRAKLLFEC
LLPFRQQVILKLIMCIEKATKT

>HmelOBP17

MTSLQLVFLVFGVVAVSLGSVSAFSDEERNKIYAGMLPLVLECSKDYGLTEDDLKAAKESG
SIGSINPCLMACVFKKINVINDKGLFDVDKAGELSQKFLTETDDQQKATEIIKTCASVNEKDV
SDAEKGCDRSKLLFDCLLPFKGQVKYNIVIILIIYSSR

>HmelOBP18

MTRQQLKNSSKLLKKNCMAKNDVTEDLVGDIEKGKFIEDQKVMCYIACIYQMSQLVKNNK
LNVEASIKQVDMMFPPEMKDATKASIENCKDVSKKYKDLCEASFWTAKCLYEDNPKNFFFA

>HmelOBP19

MTRPQLKKTLTIMKNQCMPKHRVTNEKVGQIEQGVFVEDHDVMCYIACVYKTAQVVKNK
RLDKDLVSKQIDILYPAEIREAVKLSTVKCIPVQYNYEDECEGIFYSVKCLYEDNPANFIFP

>HmelOBP20

MTRQQLKNSGKLMKKSCMPKNDVTEDQIGQIEQGKFLEERNVMCYIACVYTMTQVVKNN
KLNYEATIKQVDLMFPPDMKDAVKAAVEHCKDISKKYKDICEVSYWTAKCMYDFDPNNFV
FP

>HmelOBP21

MTMKQIKNTGKMMRKTCQPKNNVADEKIDPLNKGEFIEEKEVMCYVACIMKMANTIKNNK
LNYEAAIKQADMLFPDEIKEPAKEAITACRKVVDDYKDLCESSFYTTKCIYNYNPSIFFFP

>HmelOBP22

MGNSIKKDEEQHAMFRIKFYYIVIFLIIFTAFILVMAFTPLTKDEQIDKFNKMNEGVEPFRRNL
TECARQVKASMVDVENFLKRIPQTSLQGKCFVACILKRNSIIKNNKILKENLLEANKAVYGE
DSEVLSRLKLAINECSDVVANIFEICEFSSVFNDCMHMKMEHILDKVIMERRMEALGQMTGD
PDVWTDEEDELLKLVKDEL

>HmelOBP23

MQSAALLAAVFLALITFGFGQKEKPEFSEEIKEIIQHVHNECVAKTEVTEEDIANCENGIFKED
IKLKCYMFCLLEEGSLVDENDNVDYDMMISLIPEQYTDRVSKMITACKHLDTPDKNKCQRA
FDVHKCSYDSDPKVNF

>HmelOBP36

MSSVVKPHLRLESPFIQINCYPLLTIRRPRNWIRQLAVNNWQDICNERVLENPIAAMIHKSLT
AVAHSCMEKINGTEEDLEYLRKDPPFPEKSACIIVCLLKKIGVVKDDNFSKTGFVTAVTPLVF
HNRKKLEHMKNVSEKCDKEITTPEDTCQLGNSITACIFKYAPELHYKT

>HmelOBP39

ARSETEIKKWFIQQAVECSKEHPVTGEELQQMKTKHKIPESMSAKCLVACIFKRIEWIDEKG
MFVKEKAYKTSEKDYLNDQVKLDKAKELYESCSKVNSETVTDGEKGCERSNLLAICLTESA
AQVRLVKS

>HmelOBP43

KYAEIIMTCAKDYPITPEDIEQLKNSKLPDNAKCLFACAYKTSGMMDDQGKLSVEGVNKLA
HTFLADDPERLKKAEQFTDACKSVNEEEVSDGDKGCERAALIFKCSIEKAPQVINNKIEM

>HmelOBP2

MSILLCFLFLSIALIDGKTVNIPVDPDITSQLVQISADCIASNGLTEEVLKQVMEWKLENNEPT
KKLLFCFGTKLNTTDKNGHVILNEALKLAVSKKRPVFGDAIKRCNDQEGSDKYDTLFKIIIC
MRDQENIFLRF

>HmelOBP4

MAVMMVMSAVVSAMVVHQVITQYIYFKQIKNLGKDEKAMFRAHSEACLPQSQVDPKLLD
NLLQGELVEDAALKRHVYCVLLKCKVISKDGKLQKTAVLGKMAHRADSKNITKVLERCAD
QPGDTPEELAWNLFRCGYNKKAVLFDYMPAQVNDGADNDG

>HmelOBP9

MLVFILFSAHNMENKCDEDVSPTCVPSVDPIEECIEKLHVNRALVEKLKSGNSKLFDKNLKR
WLLCFFEKTCVMTPDGVLRQDVVLKDIPDQDKSKIEKITSICLYQKLHFAVDTAWNYLNCFR
EKDPKYSVIANKI

>HmelOBP10

MKTFIVLAICFVAAQALTDEQKDKLKKHKSECLAETKVDEKLVDKLKTGDFEIENEPLKNYT
LCMLVKSGLMSMDGEFKKDVALAKVPNAADKQLVGQLIDLCLANKGAAPEETAWNYSKC
YHQKDAKHSIFQ

>HmelOBP11

MKTFIVLAICFVAAQALSDEQKDKLKKHRSECLAETKADEQLVNKLKTGDFKTENEPLKKY
SLCMLIKSELMTKDGKFKKDVALAKVPNAADKPAVEKLIDLCLANKGNTPHQTAWNYSKC
YHEKDPKHSIFQ

>HmelOBP24

MRAVWCLLAVSLAVVSGKVLDTLVIVPPERVPGIVQASSKCIEEMNLDKDTMQKFFSWQLG
DSESTRKYMYCLGVKSGYIADDGSMVKKEVLGLAGSHGGNIDGVIDECNNLKYSDKYEAV
FKIVMCFHEKSKLEFKV

>HmelOBP25

TIILPPARAFDIVQASSKCIEELNLEKNILQKYLAWELSDCESTRKYMYCLGTQSGYIANDGSI
VKNKAFEVVGSHRNRVNGIIDECHEFKHNDKYEAVYRNVMCFNKKSRLHFKV

>HmelOBP26

IVILPPARVPGIAQASSKCIEELNLEKDTLQKFLAWELSDSESTRKYVYCLGTESGYMAKDGS
IIKNEVLEVVGNHRNRVDGVIDECNERKYNDKYEAVFHNVICFNEKSGLHFKF

>HmelOBP27

MDTGKMRAVWCLFLVTLATVYAGNVKVEYVDIPKDYIPAVEKASFECIKKLKLEEEQISLQ
GFLNWELSESDNTKKYIFCLANGSGFFADDGSILKDKVLAIMGKYRDRVDKVIDECSKVKY
DNKYEEVYRMEVCFRDLSGLYFRM

>HmelOBP28

MNTLWFFLFLSIALVKGKALFHVPPEYAGEILKAAADCIDSTGAGVDAVQKVISANLENTEP
FKKFLYCFSSKSGYVDSDGHFIVDQMTKLIGNHKDKAKFIDNLNLCNKSEGGNTIDTIYQIAV
CFKDNSPIYFTV

>HmelOBP29

MKTFWCFLFLSIALVSGRAIVNVSQEFTGDILKTAVDCTDSSGAGVDALQKMASANFEDTEP
FKKFLYCFASNSGYVDSDGHFIMDKMTKLIGNHKDKAKYVDAINLCNKRKGGRTIDTIYEL
ANCFKDHSPIYFTL

>HmelOBP30

MFDIPKEFVPDIIKASAECADKLGLDTLNILSKFFSGELKDSESVRQYMYCLGTTSGYVNDDG
RLNKDRLGKVVGEHKSKVESVVDECNKAKASDKYETVYKAVVCFRENSGLQFKI

>HmelOBP31

MLMRKNPICIYISIIYLGVQYVLVEYIKVPKEFIDDAINGSGQCAEELGLPGDTLNKLLSNNFE
DSQAMRKYIYCLGIALDVGDGTGSLKHSLSKYASNDRRKAEITKTIDECNKEKASDKYEKA
YKVSTCYLNTSSVQFKV

>HmelOBP32

TYFKIPKEFIEDAIKGSVHCTEELGLPVDTLNMFLSDNFEDSPTMRKYIYCLGLAVDVSEEDG
SLKHSLSKYANNDKRKAEVIQRVDECNKQEANDKYEKVYKVSTCYLNTSSVQFKV

>HmelOBP33

AYIKVGKEFTEDAIKGSVHCTDELKLPVETLQTFLTSKYEDSLPMRKYIYCLGIMLDVGDEN
GNLKHSLSKYAGNNKRKAEILETIDECNKLEASDKYEKALKVSTCYLNKSSLLFEIKKD

>HmelOBP34

AYIKVGKEFTEDAIKGSVHCTDELGLPVDTLQTFLTSKYEDSPLMRKYIYCLGIMLDVGEED
GSLKHSLSKYAGNDKRKVEVLKTIDECNKLEASDKYEKAIKVSTCYLNKSPLLFEIKKD

>HmelOBP35

MKVLIICVGLFALIEAHNIHLSHGQKEKVKEYAAECMKESGAKPEVLADAKKGHLVDDEGL
KKFILCFFQKTGVLSSDAKLNTDVALSKLPAGIDKVTAAKVLNDCKNKKGATHADTAFEIFK
CYYTHTKQHILFEK

>HmelOBP37

IIAMPPARISRIVMASSKCIEELNLEKDTMQKFFAWQLSDSESTRKYTYCLGTGSGYIDSDGSI
IKNEVLDIVGSHRDRVNAIIDECNKSKYNDKYETAFRSVMCFNEKSGLQFKV

>HmelOBP41

YLKESAAKSKILANTNKRHLIDYEELSSDDKLNTDIALLKLPAKVTAAKVFNDCENKQGATR
AYTAFEICNVTTLLHNQLLYLKNK

>HmelOBP42

YAGVILKAAADCIESTGVGADAVQKVIAANLENTEPFKKFLYCFSSKSGYVDGDGHLIVDQ
MIKLVGNHKDKAKFIDVINLCNKSKGGNTLDTMFQTTVCFKQNSPIYLTL

>HmelOBP1

MSVRVLFIFIIVTACQANIQVSPPVTCGYLPRAIHECIGSPHIVKPEISAQCSKSISECERMTCVF
QKSGWMSGNAVDKDKVKSYFDQFSTDNPQWALAVNHVKAACLNMDLPSQGVYLNCPAY
DILTCVFSGFIKNAQPDQWSSSESCSYPRQFASACPYCPSDCFAAQVPIGSCNACLALPRSP

>HmelOBP5

MLRIPLVAIFILKPISACCDIPEMGDVGPLSECSNPRLPGPCNEVQCVFEKSGFLIDKQTLNKE
AYRAHLRQWVEGHKGWEDAVEKAIKDCVDRDLRQYLDNPCKAYDVFTCTGIAMLKVRK

>HmelOBP38

EFIQPKWRTDCGFKLKWDDINRLSIFNESDVTTTTTEATSLRNKDVKIVPLPCDKQNCIFSKL
NITNDGVIDKDAFSRLLDTMTHRHTQWTSAKAKVVTQCLNKPLLGYEEDCEINRILACTYDI
LTEVCY

>HmelOBP40

MHQTVVFCLLIFQVVTAEPPPPQCRGPPAGLQKNPKECCKFPKVFKEEDFKECGIEKPSEEDG
SFHHRGPPDCDKQICLLQINNLMKDDTTIDKDAVTAFMQKWGDANGDFKDAVDVAIDRCV
KGDLPGPPELCEATKMIFCIGSTMFMNCPKWEDTDDCKKVKEHIEECKPYFN

>HmelOBP45

MVQRISALLWCLYIFSISLSDSAISAETETRCRNPPTAPQKIERVISLCQEEIKLSILREALDVIK
EEHTMPAQRRRNKREVPFTHDEKRIAGCLLQCVYRKVKAVDGYGFPTLEGLVGLYSDGVN
ERGYFMAVLEASRECLMRNHDKFSRTVPMDNGRNCDISFDIFECISDRIGEYCGNSGL

>MsexGOBP1

MGQNTRSLVLVVLVGLVGAVSADVQVMKDVTLGFGQALEQCREESQLTEEKMEEFFHFW
REDFKFEHRELGCALQCMSRHFNLLTDSSRMHHENTDKFIKSFPNGAVLSKTMVELIHNCEL
QHDAEEDHCWRILRVAECFKISCTKAGIAPSMEVMMAEFIMETENK

>MsexGOBP2

MVNRLILVVVVVFITDSVMGTAEVMSHVTAHFGKALEECREESGLPVEVMDEFKHFWRED
FEVVHRELGCAIICMSNKFELLQDDTRIHHVNMHDYIKSFPNGQVLSEKMVQLIHNCEKQYD
DIADDCDRVVKVAACFKKDAKKEGIAPEVAMIEAVIEKY

>MsexPBPA

MKVAVVAIVVYLAVGNVDSSPDVMKNLCLNFGKALDECKAEMNLSDSIKDDFANFWVEG
YEVSNRDTGCAILCLSKKLDMIDPDGKLHHGNAMEFAKKHGADEAMAKQLLDIVHNCENS
TPPNDDACLKTLDIAKCFKKEIHKLNWAPNMDLVVGEVLAEV

>MsexPBPB

MKEAGVRFKILLFLIFPVVTGNFKGKQIMRSVAETFGRTVFECQNEVLMKFGSGILNDIFRY
WHEGQPLEDRDLGCIFRCILLKLELVNDNGRLIDANADGFFQANGADESMTKHLIELYHSCY
QTMRFPQDDCMLILEIGKCCREGVRNAHWTPGSK

>MsexPBPC

MAVIPIFTVLLMMTAVKEIAPSSDAMRHIANGFLKVLDQCKHELGLTDQIVVDLYQFWKLQ
YALLNRDTGCAIICMSKKLDLLDGTGRMHHGNTQEFAVSHGATDEVASKVVVIIRDCEKQQ
EGEQDDCVRVLEVAKCFRTAIHELNWAPNMEVVVDELLTEI

>MsexPBPD

MVSTKWCFLLVTIAVLTMEVVSASQEVLKQMSVGFSKVLQTCKTELSVGDHIIQDFYNYWR
EDYDLLNRDFGCMVICMAVKHDLINDQLTMHHGNAHAFAKTHGADDDTAQQLVTILRECE
AKHQSVEDVCNRALEMAKCFRTKIHELKWAPAMEVVLEEIMTSV

>MsexABP1

MSVISFFVLCFGVLAVSVGAVSENERNQISQSILPHIVKCSQEYGVSEGQIKDAKESVNPLGL
NPCFLGCVLKSAGIIDKNGLFDVEATKEKSKKYISSEKDVTNFDKIIKDCTEVNQKNVSDGN
KGCDRAKELVTCFLAKRGDFSVFTF

>MsexABP2

MDRKDLCLLIIAFILADGVDSMSKQQLKNSGKMFKKQCMGKNKVTEDEIGEIDKGRFVEQQ
NVMCYIACIYQMSQVVKNNKLNYEASLKQIDIMYPPELKDTAKGALEACKDIAKKNKDLCE
ASFKTAKCMYEYSPKDFLFP

>MsexABP3

MQKQRGAMITATLHVVFALLGFVYGAKNKPVFSEEIKEIIQTVHDECVGKTGVSEEDIANCE
NGIFKEDVKLKCYMFCLLEVAGLADEDGTVDYDMLVSLIPEEYSERASKMIFACNHLDTPE
KDKCQRSFDVHKCTYEKDPEFYFLF

>MsexABP4

MFRYIFCAICLILILFDASYAMSRQQLKNSGKMMKKSCIPKNDVTEDEVGQIEQGKFIEDRRV
MCYIACIYTMTQVVKNNKLSYDAIVKQVDMMFPPEMRTAVKTAAENCKDIAKKYKDICEA
SYWTAKCMYDFDSKNFVFP

>MsexABP5

MCKPMYYVVFSIIYLSIVLAQKADNGNTKIANVQRNDQGSMDDVDVEDIMNQCNETFRIEM
AYLQALNESGSFPVETDRTPKCFLLCVLDNTGVMTKDGDFDPERTAALFAGERAGKVMDGI
QDMAAACADRKEKCKCEKSYNYLKCLMTMEIEKYANNN

>MsexABPX

MISSLVHVLTLLAAGVLALDEEQAELARMVRENCVDEIGVDEGLLAKVDDGADLMPDPKL
KCYLKCTMEMAGMISDGVVDVEAVLGLLPDDVKLRTTDIVRACDTQKGADDCDTAFLTQT
CWQQANRADYIFI

>MsexOBP8

MCKPMYCVVFSIIYLSIVAAQKADNGNTKIANLQSNDQDSMDNVDVEDIMNQCNETFRIEM
AYLQALNESGSFPDETDRTPKCFLLCVLDNTGVMMKDGDFDPERTAVLFAGERAGKVMDG
IQDMAAACADRKEKCKCEKSYNYLKCLMTMEIEKYANNN

>MsexOBP9

MYVLKFFACIVLIGCIKAKTLDDLKNNYLEMIVKCTSKYPITDEDLELLKTGDMGDKESTKC
LFACVYKKTGMMDDKGMLDVDQTNKIVQTYFSDNSEELKKGLAYTEACKSVNDAPVTDG
DRGCDRAALLFKCTTENLPALGGLAVKYCFFLELNDETIMPLA

>MsexOBP10

MIRAVVFCCCMVALMPFSANAMTDEQKEKIHEHFEKLGLGCLKENTITEDDIKDLRAKKVP
SGENAGCFLACMMKEIGVLNDEGMLEKGRAMELAKEVFDDAEELKKIEEYMHSCSSVNSE
SVGDGEKGCERAMLAYKCMVENASKFGFDIVTNILHSKLILSYLNINLHSS

>MsexOBP11

MWKISYFLFVFGVAANLKHAYAVTEEERLMLDSLMKPKVLACVEEFGLKDFSIEDIRKDHEI
DPCLLQCFLKKAEVFVDGMINLEKADETLREVINDEDEVEQIMEKGKECADEANGSDVSGD
DEDCARVAIFHSCLREKNGLFMATS

>MsexOBP12

MPKHDVTEDQVGGIEQGKFLENRNVMCYIACVYSMSQAMSKQQLKNSGKMFKKQCMGK
NKVTEDEIGEIDKGRFVEQQNVMCYIACIYQMSQVVKNNKLNYEASLKQIDIMYPPELKDTA
KGALEACKDIAKKNKDLCEASFKTAKCMYEYSPKDFLFP

>MsexOBP13

MLFLVVAKFFILLSLGEAMTMKQIRNTGKMMRKQCQPKNNVEDEKIDPLGKGVFINEKEVK
CYMACIMKMANTIKNGKINYEAAMKQADLLLPEDIKEPAKEALTSCRKVADSHKDVCDAA
FYISKCIYEYNPDIFYF

>MsexOBP14

GITEKQRLHIREELIEIGTECIIKNPITIGDVRSFRNKQFPNGPNAGCFIACVFNKAGIMLGLLEI
NNFPTVQTQDALLRAYLIKQELFDDEGLVSQKTATEKATKVFDDETELKNYEQFIAVCDKV
NEESVSDGQKGCERAKLAFQCLIQNSKQVFIPYNLLLGSR

>MsexOBP15

MRRKLLNIKENPFTSVVRKALINTARSCMVHVNATQEDLEYLRKDPPFPEKASCIVKCLLEKI
GVVKNNKYSKSGFMMVVTPLVFANKKKLDHMKTVSENCESEIVHKESSPCDAGNEVTTCIF
KYAPELHLRG

>MsexOBP23

MYAHDKLSDMVAEQCLNEMYPKSKRIEIQESDEPCIIFCVLKKFGIMSTNGVINLEVFRKRV
QNSHQHEQRNTMNDIGSNCLESAEATQHKQDVCKKAKVFNDCTHLYRILLK

>MsexOBP24

MLVLKFLLCALTSALAMKQHAPSGTMVDFTDAKVQGHLDALVRMAQSCVIKVRATPKDV
RAYFTNSSPVSRSGQCFAACMLEQSDVVNHGKINRELLVHLASLVNGKNSRVVRKLNSVSR
LCLDSIDGMSDRCQLASTYNDCLNENMIEFAFPLDIAEEAVRKMPFHLIQPKNLPQEMRPAN
Y

>MsexOBP27

MKRAKRRQPRQETNMFKIDFQSLMLFLIFGAAFILVIAFQPLSKEEHIERYNKMSEDVEPFRK
NLTECARQVKASMVDVEHFLKRIPQSTMQGKCFVACILKRNNIITKNKVNVKNLLEANRAV
YGDDSEVLARLKTAVKECSEVVEGVFEICEYASVFNDCMHMKMEHLLDRMTMERRMEAL
GQMTSNPDEWSDDEDEMLKLVKDEL

>MsexOBP29

MMGVDAIHDPQTKIDKDTIITRNLKLEKKGQAKTSVQNRKKDIEREPDWSYQPIPPEVSTHV
EQFKKNMTECLKEVQANDKRPVKRLSPKTESPIHGECLIACVLKRNGVIDNGKINKNNLLTL
VSKFYAKDTKLMKKLDKNLEHCIEISTRNRDECVLASQLNACTNDLMASNKHKIIVNY

>MsexOBPY

MPKHDVTEDQVGGIEQGKFLENRNVMCYIACVYSMSQAVKNNKIMYDNMIKQVDMMFPP
DIKDAVKDSIENCRPVAKKYKDVCEAAFWTAKCMYDYNPANFVFP

>MsexABP6

MKVTNGLFLVFLVSVLACGIAASPKKIYRIPPQSSEKIVEEVLKCVQKMGLESTVVNLLKEG
KYTEDDRVIETLMCSNQNLGNVNGDGKVNIDKVMNEIFSNKPEIRSALVACEKDGGKSSLET
FKNX

>MsexABP7

MQACLFLTLVLAVVGLNAHNVHLTDGQKEKANEHIAACIKETGIKPEVIAEAKKGHYSEDE
AMKKFILCFFHKAGIVNADGKLNLDVAIAKLPPGVDKTEATKNLEGCKDNGGKDAAETAFA
IFKCYKDATKTHVLF

>MsexABP8

MKALLVLAACLVLAQALTDEQKEKLKKHKSECLSETKVEEQLVNKLKAGDYKAENDNLK
KYALCMMMKSELMTKEGKFKKDVALSKVPNPADKPMVEKLIDTCLANKGNTPHQTAWNY
VKCYHEKDPKHAIFL

>MsexOBP7

MYFKILVLCGILAIKNGICDERRAVSFVPEVADHITKTIKYCMVKHDNDPKIIELVRQGSYGV
DEPFKKFIHCAYYKSGYANEDGHVLVNKVIKAFPKDANIEEVTKKCSTIKGEDAEDTTYQFF
KCFELNAPIRLALE

>MsexOBP16

METFQFAVFCVAFYTFVVSSPIKEFYPKRDVATLKKYQMECVEDTKVDPDLVIRFKAGDWR
SEQPSLKNWVLCILNKMGLMTMDGVYRLDEAMARVGTKDKDMAEKLIDQCLSTTALPAPD
IAWKYVHCLHVNDPLGNYSSISILTP

>MsexOBP17

MQSCLFLTLLVAVVGINADTVLLTDIHKEKSNANIAACIKESGVKPEILAEAKKGNYSEDEA
MKKFLLCFFNKSGIMNADGKLNLDVALANLPPGVDKNEATKALEECQHKNGKDAPDTAFTI
FKCYRAATKTQVLF

>MsexOBP18

MYQYNGYRSSSQSPRRYKRERRVDNTGQRSQYNPNTQRNSGYEDTYRNEEKNSSENNTNT
DNKACALQCFLENLQMTAQDGMPDKYLVTHAITKNVKNDDLQDFLQESIDECFQILENEDS
DDKCEFSKNLLLCLSEKGKANCDDWKDDMHF

>MsexOBP21

MIKQMCAFLFALSVICSVNLAASKSIYVFPPEKAEMILENAIKCITESGLQTFVGQEIKQGKYT
DDERTLGALVCANEKIGYSNESGRLNIDKIMIDLFPLKPEIRSDLEACNKDYGLDPVGTFKSF
LICFRKRVPFRVVL

>MsexOBP22

MSWLHAVVALALAASAAPATTSCKNCIALGKEEKAMFRAHSDACLPQSGVEPKVVESMLN
GQLVESAALRRHVYCVLMKCKLVSKEGKLMKNAMLGKMAMRSDGKNATKVLEGCADQT
GDTPEDLAWNLFRCGYDKKTMLFDYMPTSGASSGDIDNISK

>MsexOBP30

MYINMDFRVKLVTRCLTLKRHSYCKMKITASLVFVLLNVVSIYGESKEIYFFPKEAAVTFLE
ATLKCIADSNYDSSVIDQIMQGKYIEDDKTVNALICACVATGFGYPDGKVNVEKIMKESVPT
RQDLRPFIEDCNRESGKTPAQTFRGIVKCYREKLPVQLRFSN

>MsexOBP31

MQEVEYADERRNVTDILKETDVPNSTSFNDEGFTTRNLEDAALDSTKIQIIRKYNDTDTISRR
KKRSEPLFDKPDTNQCLSQCVFANLQVVDSRGIPRETELWNLVQSAVTSQQSRAALRDQIRA
CFQELQSEAEDNGCSYSNKLERCLMLRFADRKMDEGNANEKKADQ

>MsexOBP32

MLCMHHCKLTIFVTTINMQSYLFLTLVLAVVGINADIVLLTDVQKEQSNANIAACIKESGVK
PEILAEAKKGNYSEDEAMKEFLLCFFNKTGIMNADGKLNLDVALANLPPGVDKNEATKALE
ECQHKNGKDAADTAFTIFKCYSAATKTQVLF

>MsexOBP33

MKAAQNLFGIILFSVFAAVIAASTKKIFHLSPESGEKLVEGIIKCIAKLNFDPSLINLIKEGKYLE
DERLIKAIICMNVDSGVGTADGRLNVDAVMERIFSNNAEIRKGLICCEKEYDGTPVGNLRGT
LTCLKETLPFKIRM

>MsexOBP35

MRTAIFLLLVSVISCYAAEEKDTYYYPKAPSEIFLNAAKKCVEDLSYNSSIMDQIMQGKYIEE
DKTLNVLICAAVNTGYGNADGKLNVEKVVKELYPERQDVWPIIEKCNLEQVTSTPLETFKGI
VVCLKNNLPFKIRFPM

>MsexOBP36

MKVTILVILIIGVSCDAAFTKPIYRFPPKQSEMYVEAVVKCVAKLGYDLAILDQIRQGKYTDD
DKSVEALVCANNDIGYGLPNGQLDADKTIQDLFPTKPEIKSVFDKCDKDYGVDPAGNFKAF
LLCFKDEIPFKVII

>MsexOBP37

MTKSSFFLFQDIFRFPPKKAEIIIEEVVKCHAKLGYDSSIFEQIKQGKYTEDDRTIEVLICANLN
MGYGYENGMLDVEKTMTGLFSKNPEIRPMLEECDREGPTPVATLKAFLLCFRDKIPFKIRI

>MsexOBP39

SIGASRQQWDSCLNHSIERRNTSQIFLILQELYYFAKEPSKIFLNEALKCIGKFSYDASIIDEIKL
GKYIEDVKTAEVMSCACINAGFCHKNGRQNVELAMNIIFSRRQDVRPIVEECNQDVAPAPVE
TFTAVLLCYREKLPFKTTFPE

>MsexOBP40

MRAAIFLLLVSVISCYAAEEKDTYYYPKAPSEIFLNAAKKCVEDLGYTSSIMDQIMQGKYIEE
DKTLNVLICAAVNTGYGNADGKLNVEKVVKELYPEKQDVWPIIEKCNLDQKTSTPSETFKGI
VVCLKTNLPFIIRFPM

>MsexOBP19

MKLRTKWNGIYTSSLKIVFIKYVPAKLPSLHLMVISSQGPPPFLKDIPEKCRGPPPVVEKPHEC
CKVEPFFEASDFTECGYKNSNEDVGFKRGPPDCSKQLCLMKKYNLAKDDQVDFEALKKFLD
DYAEKYPAFKSAVEKAKECVKEDLPGPPSVCLANRIVFCIGSVVMFECPAENWSETEGCKTL
KDHMTECKPFFQRQ

>MsexOBP20

MATDKEGINADLLRQDRSKATLKPISACCDIPELGDAKPLSECSNPKLPGPCKDVQCAFEKS
GFLTDNLTLKKEVYKKHLRQWAKNHDGWSDAVEKAISDCVDKDLRQYLDIPCKAYDVFTC
TGIAMLKKCPDAAWKC

>MsexOBP25

MQTLRETRPRCTAERFLIMFKIVLICAFGIAASNADAPGDPPKVYCGEIPYKIFECLGSPRIIKP
ESSMKCDKSISECDKMRCIFREEGWMVNNVVDKSKVGASFDQFGKDNPDWAAAIGAAKTE
CLAQELPAQGVFIGCPAYDILHCTLTTLIRNANPAKWSSSEECKYPRQYAGACPICPSNCFVP
AIPTGSCNACLSLPRTP

>MsexOBP26

MEKNLPLERRPVWFLKNYFKTKCCRIPPFINTTILTECGFSSLMQYYTHAPQYGTTIAYPMVH
FAPIQAPPTDEKDKVHVVDLNDVTEAPEDVTDPLECCNVDEFIKPAWRSECQFELTWNDQS
RLSIVDKPAATTPAPSTTHRPARERDIMVVPQSCEQETCVFKKLNIISDSGAVDMDAFLKLLD
NFTDAQPTWMKAKARVVTTCLTKPLRDYDAECDINNVLACTFDVLTEKHRRDTCFLPEIVS
REVLTECGLKSLTRVEQAPETPVVNRKKTFALAKYSCIDQTPPTGCLMGKMGVLSRYGFMD
HFQMKDRIRKFSNGFDEWKPWTDVYLGAFTNIPLYRDYCNSPKKLLNLLDAMIMTCPVSRR
KDTPQCAKVFTEMTHSIPVHEQNITKVKLDRILNHFHHVFWPVSTQGNHASQKHYKIVKKPI
YDYGILKSKNEPKVQIIDVKPVPKKPLVLVPVYLRLPAQNNLSRPYANDGVWRSNPFWLHT
QIALAHLNTTTTASANPTIATKTTTPTA

>MsexOBP28

MYRLALLLVLSCVLLVQADDEWMDFMQKCGQLIHPAGIRCCKKKYVSTDLTEDLKECMQL
PGKPPSCEIEICVAKKRGFASDDGTINKAAFKALLSKMDDKDLQASIVEDCVDGDISEYGPD
DLCDLKRLHTCVALHLVVDCPEWSNEGACSGSADVAADCIKSMQ

>MsexOBP34

EQRCRNPPTAPQKIERVITLCQDEIKLSILREALDVIKEEHTMFLITEALDVIKEEHTMPAQRK
RNKREVPFTHDEKRIAGCLLQCVYRKVKAVDGFGFPTLEGLVGLYSDGVNERGYFMAVLE
ASRECLMKNHDKFSRTTPMDNGRNCDISFDIFECISDRIGEYCGNAGL

>MsexOBP38

MNFIQGNITGFTRRTISRCKHWRCVLSKYDMLTPTGRLDDEKYYIHLDKWVELNPSFANAM
LNAKVNCKLSFRHVMPLDPCEFYNFHGCIRNYIDLNCPAYVNTPQCAEVKEFHAECREFFYK
K

>BmorGOBP1

MWKLVVVLTVNLLQGALTDVYVMKDVTLGFGQALEQCREESQLTEEKMEEFFHFWNDDF
KFEHRELGCAIQCMSRHFNLLTDSSRMHHENTDKFIKSFPNGEILSQKMIDMIHTCEKKFDSE
PDHCWRILRVAECFKDACNKSGLAPSMELILAEFIMESEADK

>BmorGOBP2

MFSFLILVFVASVADSVIGTAEVMSHVTAHFGKTLEECREESGLSVDILDEFKHFWSDDFDV
VHRELGCAIICMSNKFSLMDDDVRMHHVNMDEYIKGFPNGQVLAEKMVKLIHNCEKQFDT
ETDDCTRVVKVAACFKKDSRKEGIAPEVAMIEAVIEKY

>BmorPBPA1

MSIQGQIALALMVYMAVGSVDASQEVMKNLSLNFGKALDECKKEMTLTDAINEDFYNFWK
EGYEIKNRETGCAIMCLSTKLNMLDPEGNLHHGNAMEFAKKHGADETMAQQLIDIVHGCE
KSTPANDDKCIWTLGVATCFKAEIHKLNWAPSMDVAVGEILAEV

>BmorPBPA2

MKLQVVLVVLTVEMVCGSRDVMTNLSIQFAKPLEACKKEMGLTETVLKDFYNFWIEDYEF
TDRNTGCAILCMSKKLELMDGDYNLHHGKAHEFARKHGADETMAKQLVDLIHGCSQSVAT
MPDECERTLKVAKCFIAEIHKLKWAPDVELLMAEVLNEVSWKS

>BmorPBPB

MKQRLRVLLLRFCILQTVLSESGVDVVKNLSLSFARFFLECDEERHFQPEVRLKVMTFWYSE
SSTWDRDVGCAFLCIFKKMEIDNPQDPSYRTHLELLSFANSEDNKIANQMVEIFYACGENTE
TDPCLWALEQVKCYKNRINQLGLTPTF

>BmorPBPC

MARYNIVVAVLVLGVVGARGSSEAMRHIATGFIRVLDECKQELGLTDHILTDMYHFWKLD
YSMMTRETGCAIICMSKKLDLIDGDGKLHHGNAQAYALKHGAATEVAAKLVEVIHGCEKL
HESIDDQCSRVLEVAKCFRTGVHELHWAPKLDVIVGEVMTEI

>BmorOBP7

AVTEEELKIEFTKLVMKCTKDHPVDMSELMQLQQLIAPKKTESKCLLACAYKLNGVMTSQG
LYNLEHAYKIAEMSKNGDEKRLENGKKVADICVKVNDVEVSDGEKGCERAALIFKCTLENA
PKVFKFGSSEYNCQ

>BmorOBP8

MLRVVVICVCFLVIAPYGINASSLDDLKMVYKNVIKECVGDYPITAADLKLIKARQIPNDDIK
CVFACAYKKTGMMTEEGMLSVEGIKDMSQKYLSDNPEQLRKSKEFAEACSSVNDQQVSDG
TKGCERAALIFKCSTEKITNFGFEL

>BmorOBP9

MLRVVVICVCFLVVAPYGINAVSYEQKIKIRDQLDRAGFECFKDHKITEDDIKNLRANKPAT
GENVPCFIACVMKKTGVMNDQGVIRKGPVLELAKKVLADDKDIKKLQDYIHSCSHVNSETV
HDKGKGCEFAMQAYTCMSANASKFGFNI

>BmorOBP10

MLRVVVICVCFLVIAPYGINAVSDEQKIKIREQIDKSGFECFKDHKITEDDIKNLRARKPATGE
NVPCFIACVMKKTGVMNDQGVIHTEPVLQLAKKVLTDDKDIKKLQDYIHSCSHVNSKTVHD
KGQGCEFAIQTYTCMSANASKFGFDV

>BmorOBP11

MSANSFVVLAFCALAVGVNALTEEQKAEITKSSLPLIAECSKEFSVNQGDIDAAKKLGDPSG
LNSCFVGCFMKKAGIINASGLFDVAATIEKSKKYLTSEEDLKAFEKLTETCAPENDKPVSDSD
KGCERAKLLLDCFVANKGSFSVFSL

>BmorOBP12

MTSFMVFFVLSVLTLKYSDALTDEQKNKIQSKFIEIGAECIVEHPISIDDINSFKNKKFPSGVN
AGCFVACIFNKIGLFDDKGNLSHNSALEKAKGIFNADEEVKNLEEFLNRCAKVNGEAVGDG
VKGCERAKLAYNCLIENSLEFGFNIDF

>BmorOBP13

MLKIHVLLCFGMAILYFGSAKAVTPEESKAFEAFAKPVIEQCQKDFGMDKESFAQKNLDEID
ECLIACVVEKFGITNDEKIDGDALKALVTKFVGNEEERNKINKIVEECTEDANKSGDGTCNTS
TILFLCLLKNGKDLWGF

>BmorOBP14

MERKDFYLLIVVVALTSGVSSMSRQQLKNSGKMLKKQCMGKNDVTEEEIGDIEKGKFIEQK
NVMCYIACIYQMTQIIKNNKISYEASIKQIDLMYPPELKESAKASAGRCKDVSKKYKDICEAS
YWTAKCMYEDNPKDFIFA

>BmorOBP15

MFLKNIFIECVLLYFVMLNTSFVNTMTKQQIKNSGKILKKACISKNDVTEDQISDIDKGKFIED
KNVMCYIACVYSMSQVVKNNKFVHDAMVKQVDMMFPTEMRDAVKASIANCRGVAKNYK
DICEASFWTAKCMYEFDPANFVFA

>BmorOBP16

MRISFLFLISVTIITFDSVFAMTRAQVKKTMTIMKNQCMPKNGVTEDQVGKIEEGIFLENHNV
MCYIACVYKTIQVVKNDRLDKDLISKQIDVLYPQEIRESTKKAVGDCINLQEKYDDWCEGIF
RSTKCLYEKDPANFIFP

>BmorOBP17

MTRQQLKNSGKIMKKTCMPKNDVTEEEIGQIEQGKFLEQRNVMCYIACIYTVTQVVKNNKL
SYDAVIKQVDVMFPAEMRPAVKAAAENCKDISKTFKDICEASYWTAKCMYDFDPKNFVFP

>BmorOBP18

MILIVIAKFLILISLCETMTMKQIKNTGKMMRKSCQPKNNVDDEKINPINDGVFIEENEVKCYI
ACIMKMANTMKNGKLNFEAAMKQADLLLPDEMKEPTKEAIVACRKVADSYKDVCDASFH
VTKCIYNHNPSVFFFP

>BmorOBP19

MTSAKTDVEIKAWFLGQAVECSKDHPVTTEELRMHKHELPDSKNAKCLMKCVFRKCNWL
DSKGMYDINAAYASSTKDFSDDKTKQENANKLFDTCKSVNEENVGDGEEGCDRSLLLAKC
LTKAAPQVSIYYS

>BmorOBP20

MAVHIFLILASYMALAAHGQLDDEIAELAAMVRENCADESSVDLNLVEKVNAGTDLATITD
GKLKCYIKCTMETAGMMSDGVVDVEAVLSLLPDSLKTKNEASLKKCDTQKGSDDCDTAYL
TQICWQAANKADYFLI

>BmorOBP21

MITASLHVIFALLAFVYGGKDKPVLSEEIKEIIQTVHDECVGKTGVSEEDITNCESGIFKEDVK
LKCYMFCLLEEAGLVNDDGTVDYEMFTSLIPEEYFDRATKMIFSCKELDTPDKDKCERAFEV
HKCSYEKDPDFYFLF

>BmorOBP32

MYSHKYLNDFTNIPEILIILLSSVALMSYGYNTKLFSHSLGSEPSLSILYARDKKSDKVTNECL
MEMYPKNLYKYPLRIDRNDIPCIIHCVLKKFGIISNDGFINIKNYYRRVQAIHRYDPRILISDVG
ETCAQNINGMNLDHDVCKKAKVFNDCTQLYAISYREPEDW

>BmorOBP33

MYAHDKLSDMIADQCLNEMYPRSKRLEIEESDEPCIIFCVLKKFGIMSPTGVINLEAYRKRVQ
LPEQLAQRNSINDFGSACLESAEATQHKQDVCKKAKVFNECTHLYKILLK

>BmorOBP34

MEKMILLNVFAVVLPCVLASRTRGSSGTLVDFTDPKVQGHLDALVRMAQSCVIKVRATPKD
VRAYFTNSSPVSRSGQCFATCMLEQSDIINHGKVNRDLLVHLAGLVNGKNSRVVRKLNSVS
RLCLDSISGMTDRCQLASTYNDCLNENMIEFAFPLDIAEEAVRKMPFHLIQPK

>BmorOBP35

GMSTHVLDFKRNMTECLKEVQNNDKRPIKRLSPKQESPIHGECLIACVLKKNGVIQNGKVN
KDNLMALVSKFHAKETKLMKKLEKNLDRCINISVKNHDECSLASQLNDCTNDIMASSKQKI
LFNY

>BmorOBP36

MAVSEISRILTFLTIVSFIYIVYSFKPLTKDEHIERYNKMNEDIEPFRKNLTECARQVKASMAD
VEKFLKRIPQSNMEGKCFVACILKRNSLIKNNKLSQENLLEVNRAVYGDDSEVMSRLKTAIL
ECSKIVEDIFEICEYASVFNDCMHMKMEHILDKITMERRMEALGQMSSNPDEWSEEEDEML
KLVKDEL

>BmorOBP37

MFYPFRFTLLFYGLFVIYLVRAEPEKENHFTLALKKTLFSTARSCMSHVNANETDLEYLRKD
PPFPDKAACIIKCLLEKIGVVKNNKYSKMGFLTAVSPLVFTNKKKLDHYKSVSENCEKEINH
DQTTECELGNEVVSCIFKYAPELHFKT

>BmorOBP38

MANLVLLLTFVLMTLSMARLKSTEAPKSKTALFNDQDNMGYEELDMEEIMSACNESFRIEY
AYLESLNDSGSFPDETDKTPKCYIRCVLEKTEILSENGVLNPATAALVFAGERNGKPMSDLEE
MAVACADRHEKCKCEKAYNFVKCLMYMEIDKYEKKN

>BmorOBP39

MVRKISALLCCFCVLGISMCDSAISTDNEQRCKNPPTAPQKIERVITLCQDEIKLSILREALDVI
KEEHTMPAERKRNKREVPFTHDEKRIAGCLLQCVYRKVKAVDGFGFPTLEGLVGLYSDGVN
ERGYFMAVLEASRECLMKNHDKFSRTTPMDNGRNCDVSFDIFECISDRIGEYCGTSGL

>BmorOBP22

MLKVFVVVVCTLGASQLCAALYTQKVAVSFPKDKTTIVVEAMKSCIAKTGANPNVIEVISSG
KVSEDEKFKEFFYCACNDIGVVNPDGHIKVKECIELFPKETQPLVEPVIKNCDKEGVNKYDT
LFKYLKCFQETSPVRVTLA

>BmorOBP23

MTSKVLLSCVVLAVLATTVLAEDSRKLVSFAPEVAKKLKVLIQECLNENGLGEDAIEVIRAG
EYREDEPFQNLVYCAYKKFGALDENNRIISQVAAASFPKDIDVVTVIESCGKEDGNTPVEQV
FKYFKCFQKNSPVRMQLY

>BmorOBP25

MKSVVLICLAFAVFNCGADNVHLNEDEREKANWYTAECGVETGVSTEVINAAKIGKYSKD
KAFKKFVLCFFKKSAILNSDGTLNMVVALAKLPSGVNKSEAQSVLEQCKNKTGQDAADKA
FAILQCFHKGTKTHILF

>BmorOBP26

MKSVVLICLAFAVFNCGADNVHLAETQKEKAKQYTSECVRESGVSTEAINAAKIGKYSKDK
AFKNFVLCFFNKSAIFNSDGTLNMDVALAKLPPGVNKSEAQSVLKQCKNKTGQGAADKAFE
IFRCYYKGTKTHILF

>BmorOBP27

MKSVVLICLAFAVFNCGADNVHLTETQKEKAKQYTSECVKESGVSTEVINAAKTGQYSEDK
AFKKFVLCFFNKSAILNSDGTLNMDVALAKLPPGVNKSEAQSVLEQCKDKTGQDAADKAFE
IFQCYYKGTKTHILF

>BmorOBP28

MLKVFIVTFFAFQLSAIARLQANGCVAVPFPKDKTIIIVEAMKSCIAKTGANPNFIDVIRSGKV
SEDEKFKEFYYCTCNDTGFVNPDGHIKVKECIELFPKETQPLVEPVIKNCDKEEGVNKYDTLF
KFLKCFQETSPVRVALA

>BmorOBP29

MTGPAAAAVLLALLAAAGQATTGCKNCVILGKEERAMFRSHSDACLAQSRVEPRLLESMM
NGELIDDAALRKHVYCVLLSCKMIGKDGKLLKAAILGKLAARPAGRDVTKVLEACAEQPG
ASPEDVAWNIFRCGYNRKAVLFDYMPAGGASSGNTENHP

>BmorOBP30

MRSFVILLNYGLLCCGQFMAEDYYYDIVTRDPDDLMREKENEVRALRAFQADCAEDVQVK
PDLVVNLKSGDWQTEDVSLKKWALCVLMKLGLMTAQGVFKMNEAMSKIPDMNDKIIAEK
LIDDCLSLQATTPHDAAWNYIKCHHQKDPEGNFSSLNIF

>BmorOBP31

MKTFIVFVVCVVLAQALTDEQKENLKKHRADCLSETKADEQLVNKLKTGDFKTENEPLKK
YALCMLIKSQLMTKDGKFKKDVALAKVPNAEDKLKVEKLIDACLANKGNSPHQTAWNYV
KCYHEKDPKHALFL

>BmorOBP40

MSEFIQPSWRTQCNFRLNWDNRNRLSIDISHGAATTQTPVPTTKPKALRDFMVVPQSCDKTT
CVFKKLNIVSDKGVVDVKSFIKLLDKFTNSYPVWNSAKARVITTCLRKSLIAYDGGCELNNI
LACTFDVLSENCPLNGNNQTC

>BmorOBP41

MLTILFLLPIVVGVLSGNIPEQPRVYCGELPNTIYSCLGNPKIIQPEVSEKCNKPISECDKTRCIF
KESGWAKNNVIDKKKVSDYFEQFAKDNPDWSAAVQNFKTTCLSDSLKPQGVDTNCPAYDII
HCALISFIKFASPSQWSTSEQCVYPRQYAGACPVCPERCFAPSVPNGSCNACLALLRTP

>BmorOBP42

MMGYACVFVILAVLQAISAEDPPGLPPFLKDAPEKCKSPPRVKNPNECCISEPFFKEADFIECG
IEKPGSERGPPDCSKQNCLLKKYNLLKNDETPDIEAIKSLLDKYIEKNPSFKSSVEKAKECLRE
DLPGPPQICLANRMTLCIGTVLLMECPDEKWNTTDDCKAFKDHMTECQKYFPK

>BmorOBP43

MKVCVLFAIFTVAQAAKATLKPISACCNIPELGNPEPLAECSNPKLPGPCKDIQCVFEKSGFLT
ENKTLIKEAYKTHLRQWAKEHEGWSVAVEKAISDCVDKDLRQYLEFPCSAYDVFTCTGIAM
LKKCPNEHWTC

>BmorOBP44

MSRLVLFFTILVVLQEFIINLYFNFITEIDSCCVKKYPKLFDSEFITECYNTQRKANDKCERDM
CVARKLNLLTEEDSINKDALLRFVEEGFKTEIDLVNAIKKKCFEEDISNIGKPEMCEVAKYKI
CITSRMAEDCPKWDSKGICSSAQQKVENFMKMLS

>VcarOBP1

ENLIEANKAVYGEDSEVFSRLKTASVECNHVIEGIFEICEYASVFNDCMHMKMEHILDKVTM
DRRMEALGQM

>VcarOBP2

KLHHGNAQEFAVRHGAGDDMATKLVTTVHECEQRHEVEEDPCVRALEVAKCFKDAMHQI
DWAPKIDVIITEVLTEI

>VcarOBP3

MFRAHSDACRAQSQVEPQLIESMLGGKLVDDPRLKRHVYCVLLKCKIISKDGKLQKAAVLG
KMSTRLDGKNVTKVTPL

>VcarOBP4

MYESCKTFFYKCNIKTVLFFIFFSIYIYFPTFATVVGYYYSNICFIFSVNDVNVSDGNKGCERA
ALIFKCSVEKASLFDFQM

>VcarOBP5

CSSLRLLLTIVLVLTLSVKNNRLDKDMISKQIDVLYPVEIREPVKKSVAKCILVQTNYEDGCE
GIFHAAKCLYDDNPDNFIFP

>VcarOBP6

MITMPLPAASTMLVQVMVTTMLALGAAPCVLQRACPARTPRPPSSVCMHRLAWNLFRCGY
DKKAVLFDFMPAGSNGTDENDAL*

>VcarOBP7

FHKMATYFVLVALVLVALGVTEISSNETMRNITASFVRVLDDCKKELNLSDNVLADLYYFW
KQDRALAHRDTGCAIVCMSKKLNL

>VcarOBP8

PISACCDIPELGEQKYLTECSNPKLPGPCNDVQCVFEKSGFLVDKHTLNKEVYRAHLRQWLE
GHKGWEDAIEKAIKDCVDKDLRQYLDYPCRAYDVFTCTGIAMLKKCPKDSWKC

>VcarOBP9

MSCMKDHPVDMTEALKLKQLIVPTKREVKCLLACGYKKLGNMNTDGMYDTEAGYKLAEL
VKSGKADDSKRFENAKKLVDICSKVNDETVSDGKEGCERAALIFKCSTENAVALGFKLD

>VcarOBP10

MTRQQLKNSGKLMKKSCIPKNDVTEEQVGKIEQGKFLEERNVMCYIACIYSMTQVIKNNKL
SYEAVIKQVDLMFPPDMKDAVKAAAEKCKDVGKKYKDICEASYWTAKCMYDHDPQNFVF
P

>VcarOBP11

MKAVILICIVIIASLEAHNVHLSQTQKEKVKSYTVECIKETGVKPEIIGEAKKGHLSDDEGLKK
FILCFFQKASIVTPDGKLNSDVALSKLPSGIDKIAAAKTLNECKNRKGNSPADTAFEIFKCYY
ANTKQHVLFE

>VcarOBP12

MHKILYIVLVSAALVYGKTSFVSIPPELLPNMFSRSMECVEKTGIDVESLQKIVTWKFEDTET
TRNYIYCFAKTTGYADEDGHLVKDRIMKIVGNHRSRNELSKVIDECNGLKGSSKDETMYKT
AVCFLKKSPILFTL

>VcarOBP13

YYLIMRARLGLCLYLICLVVRTLALDGEMAELAKMLRDNCLEETGADVALIDKVNAGADL
MGDAKLKCYIKCVMETAGMMSEGEVDVEAVVAVLPEELQKHADKMRACGTRKGADDCD
TAFLTQSCWQKASKADYILI

>VcarOBP14

MLPAALLVFLLITFGHGAKEKPELSEEIKEIIQHVHNECVGKTGVAEEDIANCENGIFKEDVK
LKCYMFCLLEEGSLVDDDGNVDYDMMVSLIPDKYTDRVVKMINACKVLDTPDKDKCQRA
FDVHKCSYAQDPNFYFLF

>VcarOBP15

MSVIIAIVKILAVLNICNAMTMKQIKSTGKMMRKTCQPKNNVADDKIDSMIKGVFIEEKEVM
CYVACIMKMANAIKNGKLNYEAAMKQADLLLPDEIREPAKEAITACRKIADSYKDICEASFY
VTRCIYNHNPSVFYFP

>VcarOBP16

RQFCIDSCTMKTLVVLAICFVAAQALTDEQKDKLKKHRSECLVETKADEQLVNKLKTGDFK
TENEPLKKYALCMLIKSELMTKDGKFKKDIALAKVPNAAEKPAVEKLIDTCLANKGNTPHQ
TAWNYSKCYHEKDPKHSIFQ

>VcarOBP17

MTTLTFLFTCCFIGIAFARSDEDIKKWFIMQALECSKEHPVSGDEIELMKNHKIPNHQNAKCL
VACLFKKVDWIDDKGMFNDKNAYKMSEKEYPGDQARLDNAKKLYETCMSVNEASVGDD
KEGCERAALLATCLTEHAPKMGFIIQ

>VcarOBP18

SRLSEMDRKDFSIFIILAVLAGGTDSMTRQQLKKSTKMLKNNCMAKNDVTEDMVGDIEKGK
FIQEKSVMCYIACIYQMSQIVKNNKLNYEASLKQVDIMFPPDMKEAMKSSIEKCKDISKKYK
DICEASYWTAKCIYDDNPKNFVFP

>VcarOBP19

TAPQKIERVITLCQDEIKVSILREALDVIKEEHTMPAERRRNKREVPFTHDEKRIAGCLLQCV
YRKVKAVDGYGFPTLEGLVGLYSDGVNERGYFMAVLEASRECLMRNHDKFSRTVPMDNG
KNCDVSFDIFECISDRIGEYCGNSGL

>VcarOBP20

MTSLTFVFLVIGVAVNLGNVKALSDDEKSNIHAGLLPFIAECSKEYGVTEEQIKEAKEAGQIG
TINPCLMGCIFKKVNVIDEQGLFVPEKAEEITKKFLTNEDDQQKALDIIKTCTSVNEKDVSDG
EAGCDRAKLLYECFIPFKGAFGQAR

>VcarOBP21

MFRFVILISFLLINYMVVDIAAMTADQKAMIHKHFEEIGVECIKDNVISKDDIANLKGKKIPTG
DNAPCFLACMFRKLGVLDDAGLLQKETALDLAKKVFNDEEELKIIENYLHSCSHINSEPVGD
GDKGCERSMLAYKCMIENASQFGIDI

>VcarOBP22

VFNGSDYNYTGYGAGNVGEKFVNSIPRPAIRKYKNNGNHSNAQNDRVKRNEMLINKNDND
QCLSQCVFANLQMVDSKGIPREAEMWNKVQTSVTSQQSRVILQNQIRACFQELQSESEENG
CSYSNKLERCLMLRFADRKVNATLNQQSATNSSFNRK

>VcarOBP23

LHFSFVRDKNDSSKMVKTFIKFFFYIAILLNLRAMAIDIEELKHQYVEIIMNCAKEFPISEDDM
VQLSNRQMPDNENAKCLFACAYKTSGMMDDQGNLAIEGVNKIADKYLANDPDRLENAKK
FTEACKYVNDVNVSDGNKGCERAALIFKCSMEKASLFDFQM

>VcarOBP24

MGWNTKWRLTFFACLLYQVYDVSASQEVMKKLTTGFAKALDQCKEELGLGDHIIQDFFNY
WREEYELVNRDMGCAIMCMASKLDLITDDMKLHHGNAHEFAKSHGADDDMAKQLVGMI
HECEKTHVGVADDCMKTLEIAKCFRTKIHDLKWAPSMETILEEIMTEV

>VcarOBP25

HKFYIITVYSQSIMFRIASFCLLVILQVVTSEPPPPQCRRIPRVENPTKCCNFPKIFKEEDFKDCD
IALPHETSSKPGPPDCSKQICLLKKYNLMKDDTDVDKDAATEFLDKFAESYPDFKNGVEKAK
EFCIKKDLPDSKVCKPTHMVFCISNILFMECPKWEEIDDCKQIKDYVEECKPYFENTN

>VcarOBP26

MFRFLVVVFATVVTCQADLAVTPPVLCGHLPKEVYSCLGAPKVVKMEVASQCSKDLSECER
MTCVFTKSGWMNGDKVDKEKLSAHLDQLARDYPEWEPAVQSAKTTCLTADLPAQGIHLN
CPAYDVMTCSFASFIKNAQPSQWSSSAKCKPSRQFAAACPVCPSDCFASLIPTGSCNACMSLP
RSP

>VcarOBP27

MGSPLFFAACVVILAAVPKSVRSTAEVMSHVTAHFGKSLEECREESGLTAEVLEEFQHFWSE
DFEVIHRELGCALICMSNKFSLMQDDARMHHVNMHDYVKSFPQGELLSAKMVAMIHSCEK
QYDDITDDCNRVVKVAACFKESCKKEGIAPEVAMIEAVMEQY

>VcarOBP28

GIVVYAKKSPARELNIRTMLIIILLSIIGSVFTAHSHGISGTLVDFTDPKVQGHLDALVRMAQS
CVIKVRASPKDVRAYFTNSPPITRSGQCFAACMLEQSDVINHGKVNRELLIHLAGLVNGKNS
RVVRKLNSISRLCLDSIEGMSDRCQLASSYNDCLNENMIEFAFPLDIAEEAVRKMPFHLIQPN
LPQEIRQTI

>VcarOBP29

PTLRAVVNLTITKHYIGVRAKRPVIVSAEATMLVANWVRFTYQASLILWMVALVTLGNLRV
VQGNVDVMKDVTLGFGEALQHCREESQLSEDKMEEFFHFWRDDFKFEDRELGCAIKCMSS
HFNLLTDSHRMHHENTDKFIQSFPNGEVLSKLMVELIHKCEQHHDAESDHCWRILRVAECF
KVSCQAQGIAPTMELLMAEFIMETEAR
