# Supplementary material for: Coexpression of Three Odorant-Binding Protein Genes in the Foreleg Gustatory Sensilla of Swallowtail Butterfly Visualized by Multicolor FISH Analysis
Source: Front Insect Sci. 2021 Jul 30;1:696179. doi: 10.3389/finsc.2021.696179 (PMC10926539; doi:10.3389/finsc.2021.696179)
Supplement: Supplementary file 2 [file Data_Sheet_2.PDF]

frontiers

Supplementary Material

Supplementary Data S2

Genomic locations and deduced protein sequences of identified PxutOBP genes.

Scaffold DF828142 (NW_013530507.1):

>PxutOBP11 (XP_013162714.1) 3086071..3087685, complement

MLCIVIFVLLPTLVICSSEGNIHLLEQEIGEAIKACIDNVSEESSTKRYTRSNRNTSNNYQYHQI
ENNKNSNPYNHERRNTSDMMTPNNVSTITDNDLLAYDDQYGNENFNNTGRNNYGNFYGN
NNNTFKRRNKRNDHYLNNNEDDQCLSHCVFANLHVVDSRGIPRETELWNKIQADVKSQQS
RVLLQNQIRSCFQELQNESEGNGCSYSNNLERCLMLHLSDRMKGTKSKKQS

>PxutOBP41 (XP_013162568.1) 5622201..5623998, complement

MQINLILSVILTIAVFGIQISSHPVLMHNILVHFLKPVSRCQQEMGLPDTIYNDFYNFWSEDYV
ITNNATGCAFICIAARLNLIVNGPNSHINLNTFFQYSRKRGADDQTAKRLLQLLRYCEGQSRD
ETDTCMRVVHCANCFRREIHALNWAPKVEEIP

>PxutOBP7 (XP_013162829.1) 7168427..7169682

MASSGTRWRMLAVCVLCAALRPPPASASQQLMKQLTAGFMKAMDVCRKELNLGDHIMQD
FMNFWREEYELVNRDLGCAISCIAAKLDLLTEDLKMHHDNAREFAIKHGADEDVAKQLVSII
HDCERSNEGESDDCRRVLEISKCFRTKIHQLNWAPSMEVLLEEIMTEV

>PxutOBP42 (XP_013162830.1) 7171527..7172691, complement

MCEMRTAVLLLVVAAGADHPPSSVINEISKQFGSMMLHCVQLLYPQTGYFRDVLDFWNRD
LNITGHTYLGCLAACSLFKLQLRNRDGSLNDTNITKFLHQNGAVENDAAVLLDVFKTCQNA
SSSERSACVAGLKTMICFRSHIYRLNWTPQFYRGPYENVLNGK

>PxutOBP34 (XP_013162831.1) 7173037..7174335, complement

MVLLATLLVALLSALAMLITPLDAYEPPRTVVNDISSKMGDIMVQCSRKMFPNYHVDPDMD
SFWDPNYKVQEVRLGCLAVCGMRWLQLTHSDGRINVANVRRFLTANDADPSTRWQLEQM
FVTCHQNSGFEQRTCSAGLTALRCYRTTIEQYGWAPGSY

>PxutOBP10 (XP_013162827.1) 7178616..7180281

MDMGLLHWCLSCALAGLALTAAPAAATAEIMSHVTAHFGQTLEECREESGLTMDMMEAF
AHYWSEDFGKATVQREFGCALICMSHKFSLLQDDVRLHHLNMDDYIRSFPNGELLSTKMVS
MIHECERQYDSVEDDCDRIVDVSLCFRSAAQREGIAPNLAMVEAALEQYT

>PxutOBP16 (XP_013162651.1) 7226623..7228090

MRCTAALAALAVLAAAPGALATSDIMKDVTLGFGEALQHCREESQLTEDKMEEFFHFWRE
DFKFEERALGCAIQCMSRHFDLLTDTHRMHRENTDRFIKSFPNGEVLSQQMVDMIHACEAQ
YDTEPDHCWRILRVAECFKASCQQRGIAPTMEQLMAELIMEAAER

Scaffold DF827756 (NW_013530893.1):

>PxutOBP20 (XP_013165496.1) 5333240..5334665, complement

MCVRRQLLLCTTAALLVAALCLDEEMAELARMLRESCAEESGVELRLVEEVNAGAALPPD
AKLKCYIKCVMETAGMMSAGAVDVEAVVAMLPDDMRAKTEGSLRACGSRAGADDCDTA
YSTQLCWQQANPADYFLI

Scaffold DF827714 (NW_013530935.1):

>PxutOBP31 (NP_001299721.1) 349639..351217, complement

MFKIIFFIIYIAVTCFATEETIRCGFIPNEIYSCTGLPSVVKPDISAKCRTKVNQCDQMTCIFKEA
GWMTGGKVDKKKLSEHFDEFKREFPEWSAAVAKLKNSCIDSELLPQGMYLNCPAYDVMH
CALTSFIQNTPASNWDREPGTGCTYSRQFAASCPLCPSDCFALLVPAGSCNACLLLPRIS

>PxutOBP19 (XP_013166306.1) 975043..982253

MRILLICALFQYAFAQVGFGTSYLRNSRLCHQWNCINEKLGLTDTLPPREQFTNILDNLLPAE
WHDAGLEALDTCYSNRTRRYTNTCPGQALMHCVVDNLMANCPGDKWTKEDGCNPVSAL
AGSKYMFSQSRYVNLQQNIQKERRPKWFLEHYFSTKCCDLPQLFNSTVLTECGFDTFLLYHL
HDPQIDAHLPSEHIRPLSSNHLRASISHFIPVRSHKPADTATTDFGGIEENEAVNDPLDCCDMT
DFILPEWREECAFQLSWHRHDRLVINEARQPVTDPPVITETNEPKVTDVKIVPHSCEIETCIFS
KLGVVSSGVVDTLALSRLLNNITGDGHWVRAKARADSHCLTKLATYEADCEINKVLACVL
DVLTENCPDAKKDDPCKHSNGSHNNITCQISSSKFRPRKRRQLCNVPEFVDMKILKECGVET
VVRIEHAPETPALKRGWTEGGKCKEETPSTTCIMRKMDILNKYNFIDYFKLKERLRNFCQGV
WYPMRNAYSAAYNAAPLYAQHCSSPNKLLNVLDTMLATCPISKRRRGENCMRIFSELTTSL
PGYHQEISNATLEDLLRRFQHIFLPGQIPHISPTNTRLVKYPRYLFNYGFLGSQDEPVVRVIDV
KPTPPVEKPLILLPVYQRMKGRSYNNDGIWRGSPVQ

>PxutOBP8 (NP_001299556.1) 5873119..5875481

MKYFVVFAICLVAVQALTDEQKEKLKKHRSECLAESKAVEEQVDKLKTGDFTNENEDLKK
YTLCLMVKSELMTKDGKFKKDVALAKVPNAADKPTVEKLIDACLANKGSTPQQNAWNYV
KCYHEKDPKHSILI

>PxutOBP30 (XP_013165846.1) 5877789..5879286

MQISAILICLLVIQGSQVHATFKRGILECVEESKVSPQLVLQVTSGNWDVREDYRLKVWALC
LLLKAKMMSKDGVFHLDVALTGVPENERRTVDKAIDDCLYKTIHKPEETAWHFLRCYHKN
HPKYSFL

>PxutOBP35 (XP_013166382.1) 6727940..6730360, complement

MPVRLAPAAALAALIFIINTHEANAECKNCIALDKEGRSLLRAQWEACGAGAVGAQDGAEL
KRRAYCALRRCKLLAKDGKLRKAALIHLARGLPADQAKILERCSKQTGDTPEDLAWNIFTC
AFEHKSLLNLPPRAADAPGEDTSRLTA

Scaffold DF827679 (NW_013530970.1):

>PxutOBP32 (XP_013167600.1) 1227816..1228972

MPKTKYQESRNEIDVINIMKECNESFRIQMSYLEQLNNSGSFPDETDKTPKCYIRCVLESSGV
ASEEGQFDAASAAVVLTQLNDGYDTNELIDMALQCTDREETCKCERSYEFIKCIMEKQINKI
ENSK

Scaffold DF827597 (NW_013531052.1):

>PxutOBP9 (XP_013168386.1) 2475100..2477035

MAAVYFIAFILLFVNCEVFADSTEQNEACQAVFNSTIDDKKCCEGSMWDDSGESTKCKDDA
DMQCDFFKCLAEENGLLVDDKINDKKVKELLNQWEKEYPSEKASIERVRRNCSGGKFIELM
ADSSASDDACEPLNFYLCVYINMIFECSSWKQDAQCSKMKEYTETCKKFLDI

Scaffold DF826949 (NW_013531700.1):

>PxutOBP17 (XP_013171780.1) 944600..946360, complement

MKYTGVFVLTLFTAVLCNEQKSKTQEKGDIGLDTVHNIKIDKNTVISRNLKLERKDRRNAKT
KQEEKELHWSYSGFPNEVSPHVEQFRRNMTECLKEVEAKEKKTVRRLSPKKDSPIHGECLIA
CVLKRNGVINKGKIVKANLILLVSKFFARDTKLLKKLDKNVDKCIEASSKNKDECAMASQL
NDCTNDLMASNKHKILLNY

>PxutOBP36 (XP_013171695.1) 3894674..3986823, complement

MLPKTLLAFLLLTRIEARISVMYAHDKASDVVADQCLSEMYPKGKRVEFQESDEACIIYCVL
KKFGIMNSNGVINLEAYRMFARGILMWLLPTCSLALRTSTFSGTMVDFTDPKVQGHLDALV
RMAQACVIKVRASPKDVRAYFTNSPPITRSGQCFAACMLEQSDVINHGKINRDLLIHLAGLV
NGKNSRVVRKLHSISRLCLDSIDGMSDRCQLASTYNDCLNENMTEFAFPLDIAEEAVRKMPF
HLIQPNLPQELRQSSF

>PxutOBP38 (XP_013171641.1) 4969540..4970191, complement

MNNNSNFITVMIILIFVSVFYLVVTFKPLTKEEHIEKINKMNSEVEPFRRNISECARQVKAGM
GDVENFLKRIPQATMEGKCFVACILKRNSIIKMNKIDHNALLDANKAVYGEDSEVMTRLKA
AINECNKVVEGIFEICEYASVFNDCMHIKMEHILDKITMERRMEALGQMSNNPDEWTDEEDE
ILKLVKDEL

>PxutOBP3 (XP_013171411.1) 5321588..5325285

MSENWRGENPVANVIRKTMIATAKSCMNHVNATQEDLEYLRKDPPYPEKSSCIIMCLLEKV
GVVKNNKYSKSGFMLAVSPLVLANKKKLEHMKTVSENCDKEVNHHDIAPCQLGNEVITCV
YKYAPELHLKS

Scaffold DF826938 (NW_013531711.1):

>PxutOBP12 (XP_013173035.1) 4767748..4769811, complement

MSFVKLVICFSFIGGALARTEAEVKEFFLKQSVECTKDHPVTAEEMTMLNKHELPDSKNARC
LLACVYRKTTWMDEKGMFMKENAYKLAQEKHPEDKAKLEKSKELFELCSKVNEDTFSDGE
EGCERAAKLTKCLTENAPKMGFELD

>PxutOBP13 (XP_013173036.1) 4770885..4772122, complement

MSILTALARFLVIITICEAMTMKQIKSTGKMMRKSCQPKNNVEDEKIDPLNDGVFIEEKEVM
CYVACIMKMANTMKNGKLNYEAAIKQADMLLPDEIKEPTKAAITACRKVADSYKDVCEAS
FYVTKCIYKENPDIFFFP

>PxutOBP26 (XP_013173039.1) 4773052..4774155

MNSVCIYFIINILYLWITLSNSMTRQQLKNSGKIMKKSCMPKNDVTEEQVGEIENGKFIEDRN
VMCYISCVYSMSQVVKNNKLNYEAVIKQVDVMFPAEMRDAVKAAAANCKDVGKKYKDIC
EASYWTAKCMYDFDPKNFVFP

>PxutOBP28 (XP_013173037.1) 4775198..4775994

MTREQLKKTLTVMKNQCMPKHRVTNEKVGKIEQGVFIAEHNVMCYIACVYKLTQVVKND
RLNKESITKQIDILYPQELKESVKRNVADCVEVQYKYDDPCEGIFYSTKCLYEADPPNFIFP

>PxutOBP22, 4777558..4779093

MFYRIVYFILLTQTLVIAMTRQQLKNSGKLIKKTCMPKNDVTEDQVGEIDKGKFIEDRNVMC
YIACVYSMGQAVKNNKIVFDAMIKQVDMMFPPEMKEPYKAAIEKCKGVAKNYKDICEASY
WTAKCIYEADTENFFFP

>PxutOBP24, 4780013..4781177

MGYRIFLLILISILTCGVHTMTRQQLKNSAKMLKKTCMAKNDVTEDLIGDIEKGKFIEQRNV
MCYIACIYQMTQIVKNNKLSYEASIKQVDLMYPPELKASVKVSIENCKDISKKYADICEASY
WTAKCLYDDNPKDFIFA

>PxutOBP1 (XP_013173033.1) 4783676..4785645

MSKLYCVFLFLGLAVSLRHVRALSQEDIAAIKTGLRPLIAECGKEFGVDEADIKKAKESGKIE
SLDPCLFACIGKKMGMINDKGEFDVEKSSETVKKFVTDKDEQKQILEIIEKCSSVNDEAVSDD
KGCDRAVLLHKCMEPYKDQFDFSK

>PxutOBP23 (XP_013173031.1) 4786590..4793334, complement

MYKNLYIYFCLIVIIPFKSHAMTAEQKASIHEHFEELGIECMKDYPINEEDVNNLRSKKIGNGE
NVPCFLSCLLKKVGVMDDKGMLQKETALEYAKKVFNDAEELKHIEDYLHSCSHINEETVSD
GEKGCDRALLAFNCMLENASQFGFDV

>PxutOBP2 (XP_013173034.1) 4788797..4791675

MAFFLFQVLYSSMIVTIVVGNSLGDLQKSYADLVIGCAKEFPITTEDIAQLQNKQLPDKESVK
RLFACAYKKAGMMDDEGKMSVEGALEVINKYLADDPDTMKKAIDFTNACSTVNDAEVSD
GTKGCDRAALMFRCSIDKADEFDINL

>PxutOBP18 (XP_013173038.1) 4792620..4794586

MINSNLFCIVILCFCNFVNGLSDAEAKAEFTKVVLQCTKEFPVDLMELLQLQALKMPKKQQT
KCLLACAYRKIKTFNDKGMYVLDEGYKVAELTKNGDEQRLINGRKLAEMCSVVNDMEVT
DGDKGCDRAAFFFKCFTENGQKLGFKM

Scaffold DF826933 (NW_013531716.1):

>PxutOBP40 (XP_013173305.1) 1561231..1563053

MIRLFFFVTLFILVTKADVFVQAPNRGATLKPISVCCDIPEIGDPKHLAECSNPRPIGPCNDVQ
CIFEKSGFLVDKNTLNKESYKKHLLKWLEDNKGWTGAIEKVINDCIEKDLRQYLDYPCKAY
DVFACTGIAMLKKCPNEFWKC

>PxutOBP6 (NP_001299383.1) 1569019..1572398

MFTITSLFILGTAFQISTSQGPPPLPPNLPSECLHPPHIENPRKCCQIPPIFTKEEFESCGFKEEHV
DKPRHGPPDCSKQLCLLKSRNLIKGDDKVDHEAMISFMDKWVDANPDFKPTVESAKAKCL
AQEVPGPIEICEANKIVFCISSVLFTECPKWVAGDEDCKKLRNHIDTCSKYFS

>PxutOBP27 (XP_013173324.1) 1573490..1575515

MLFYMLVLLLANQISISLCLPASSQDESQQCSQLPSIKNPRKCCDIPPVFSNENFGFCNLTDNF
QKNTTNGLPDCTKQLCIFKTLKLIKDEDRIDRDALSHFMDVWAEANPDFASIVRIGKPRCLE
KQIPETHEHCEINQFVSCIATVATIVCPKWIIKDECTSLKNHSDRCAKYFLH

Scaffold DF825946 (NW_013532703.1):

>PxutOBP44 (NP_001299080.1) 9782610..9783753

MNSFVFLCFVLSVAGIEAHTVHLPHSQKEKAHLHIAECMKESGVKSEVLAEAKKGVFEDDE
ALMKFTLCFFQKSGIISEEGKLNVAEALAKLPSDVDKKHAEKLLEECKKKTGKNHADTAFEI
YKCYYHGTKEHILL

>PxutOBP15 (XP_013176043.1) 9785415..9786641

MLYLILAICIAAVIQCNGNKPIVVLDPKLVENISENAEKCYKEVGAGSELLANLIPWNLEENE
TNGKFLLCLSKKLKCDGEDGHLNVDEFIALFESSLKEYDINIFKEILESCNEESGKNEYFTIFK
VLNCFDTKSPVSMAVQ

>PxutOBP29 (XP_013175978.1) 9789300..9790850

MLFNIFFIFGMVVAAHASIEGNKPILPLEGDLVSLITRKGEICVRESGVSPAIMHNLLPWRVEE
SEINGKFLLCLAKEMEFHDKDGKLKVEKFIDLFYQSLKSQDVDSYKKLLERCNELTGKNAY
YTVYKIANCFHTNSPVKMALHVLVY

>PxutOBP43 (XP_013176049.1) 9791539..9793098

MIYYLLLLFCLLGVNASPVLVKMPSQMVEKVKVVGSQCIKETGAPANSLENSLPWNLPENE
TNEKFLYCLCKNLNLINDEGYFNYERTMKIFATSDKKEAIEKTYNECKVLKGKDQYETTYKI
VDCFFKNAPVSLSL

>PxutOBP37 (XP_013176063.1) 9793772..9794771, complement

MKICTVLLISAICLLGITNASKTPFKMPAKFLEKSKLVGEKCVKEAGAESDFLEHLFPWNYPK
TEAYEKSLLCFATTIGLANQDGIFYTEKVHPVFSSSENFEEIKKAFAECNAMRGNSPQDTAFII
SECFFDKAPIRLTM

>PxutOBP33 (NP_001299436.1) 9797225..9798334, complement

MIYTVFLIILLPILGTYGSKPVLKMPSQSTEKFMAAATHCIKETGAPEDCLTLSLPWKLPQNEL
TEKYLYCLGQTTKLANSDGHYYPEKVMKLFAGSDIKEDIEKTMVECNSLEGDNVFDVNYRI
TDCFHKKAPVILSL

>PxutOBP39, 9804497..9805340, complement

MIYPVILFTVLCVLGANASPAPLKLPAQIAEKVKQVGSQCIKETGAPADALSNTIPWNLSENE
DNEKFLFCLCKGKNLIDDEGHFDQEKTMNIFASSDKKR

>PxutOBP5 (XP_013176078.1) 9807732..9808617

MLYFVVALSVLSVVALGDQSKPVIHLPPIVLNVAKEAASTCLKETGASQEVSDNFFKLKFGS
DPDSKNFIYCLCRKTNYADEDGHLNEALLTLFEGNEHKDAVAKVIDTCNKHQESNKIDTMY
KTVECFYKNTPVHLSV

>PxutOBP25 (XP_013176067.1) 9835865..9837028

MYFVLFCALSALTLSSAKQTYIDLPSDVVGLLTEKSVGCIAETGADINILQRVFQWQFDNDK
TSKKFSFCLFKNVGLIDDTGKFDESQIISSLYRNSNKKEEIAKIAKECNSKDIKKPLNKMHEGI
QCFFKNTPVLLQVKL

>PxutOBP4 (XP_013176038.1) 9839887..9841577

MMVKTTTLLFLCTVLVGITTARKATIGVPSDIVGLITEKSVECIAETGADVNILQRIFNWQFD
NNKTSKKFSFCLFKKAGFSDDTGHFHKDKILALYEKSDKKEEIKAAVDACDKIAEKNPLETM
YKVILCFFDKTPVLLEVKLD

>PxutOBP14 (XP_013176133.1) 11349596..11352512

MWTASLPVFFTLIVFGRTEKEKPELSDEIKEIIQHVHNECVAKTGVAEEDITNCENGIFKEDTK
LKRYMYCLLEEASLIDDEGNVDYDMMVSLIPEQYYDRVHKMIFSCKHLDTPDKDKYQRVF
DVHKCSYEKDPNFYFLF

Scaffold DF825928 (NW_013532721.1):

>PxutOBP21 (NP_001299301.1) 6955288..6974082, complement

MIRKISALVCCLCVFGISLSDSAISAENEARCRNPPTAPQKIERVITLCQDEIKLSILREALDVIK
EEHTMPAQRRRNKREVPFTHDEKRIAGCLLQCVYRKVKAVDGYGFPTLEGLVGLYSDGVN
ERGYFMAVLEASRECLMRNHDKFSRTVPMDNGRNCDVSFDIFECISDRIGEYCGTSGL
