# Supplementary material for: Coexpression of Three Odorant-Binding Protein Genes in the Foreleg Gustatory Sensilla of Swallowtail Butterfly Visualized by Multicolor FISH Analysis
Source: Front Insect Sci. 2021 Jul 30;1:696179. doi: 10.3389/finsc.2021.696179 (PMC10926539; doi:10.3389/finsc.2021.696179)
Supplement: Supplementary file 3 [file Data_Sheet_3.PDF]

*Supplementary Material*

**Supplementary Data S3**

Supplementary Figures S1–5 and Tables S1 and 2.

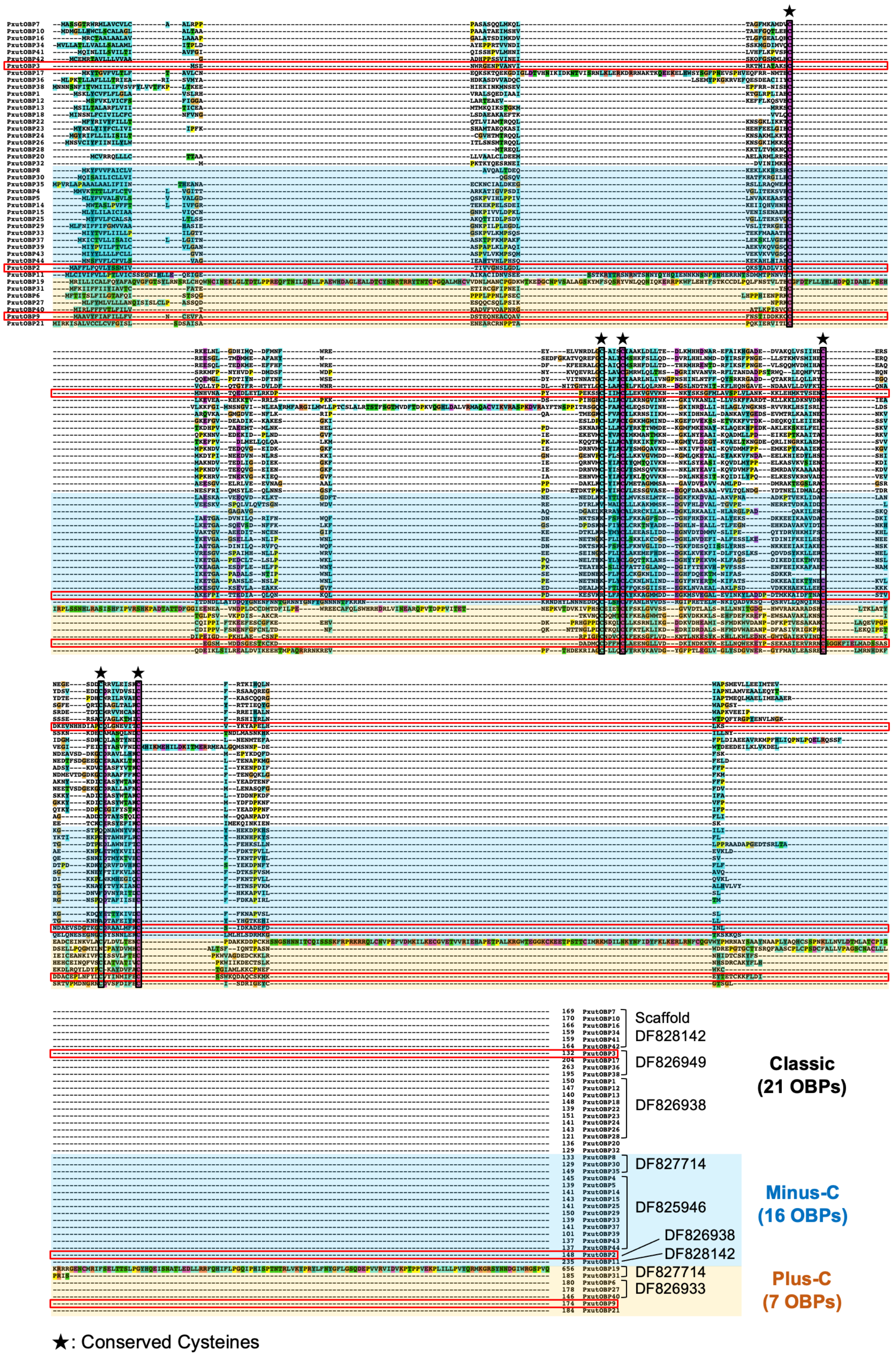


**Supplementary Figure S1.** Alignment of deduced protein sequences of *PxutOBP*s.

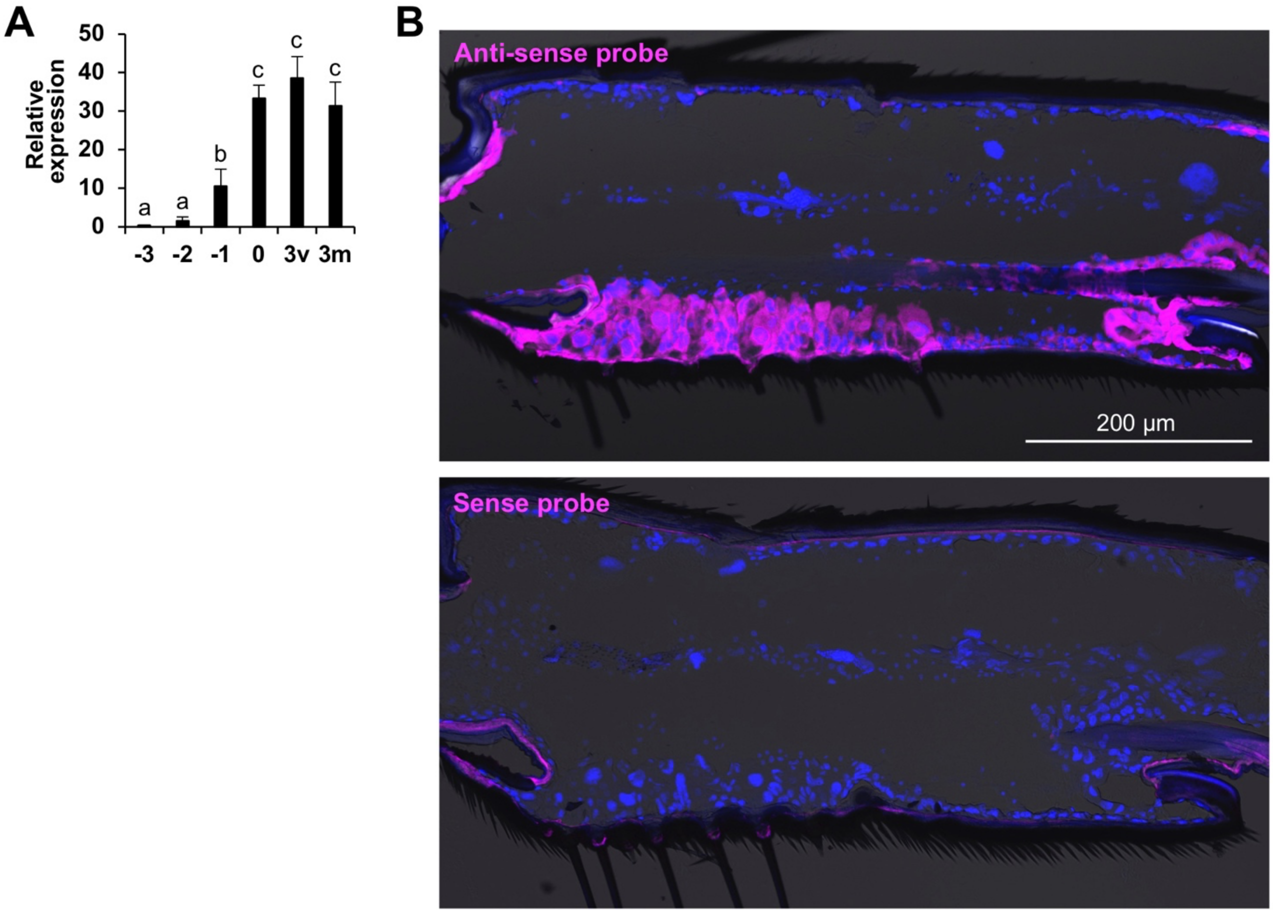


**Supplementary Figure S2.** *PxutOBP1* expression in the female foreleg tarsus. **(A)** Developmental time-course of the expression of *PxutOBP1* in the female foreleg tarsus analyzed by qRT-PCR. **(B)** Spatial distribution of *PxutOBP1*-expressing cells in the fifth tarsomere visualized by FISH. 0-day-old females were used.

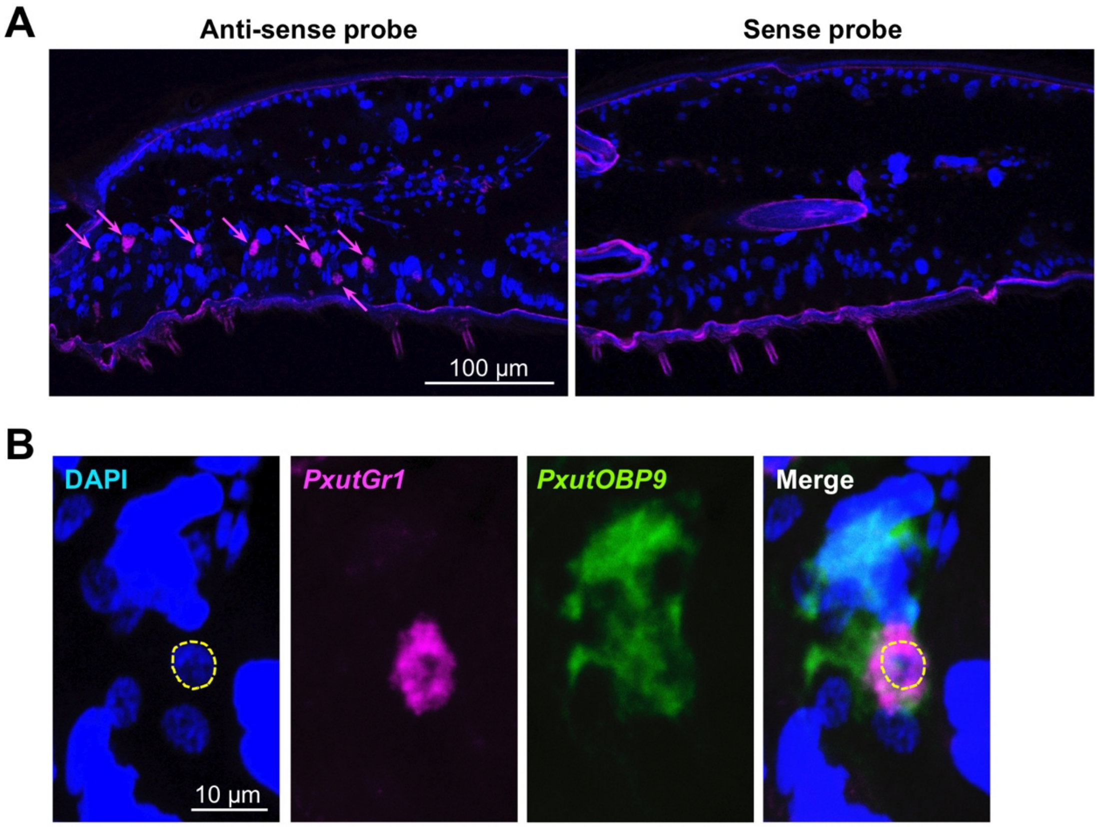


**Supplementary Figure S3.** Visualization of *PxutGr1* expression in the female foreleg tarsus. **(A)** Clear stainings (arrows) at the bases of the gustatory sensilla were only detected by anti-sense probe for *PxutGr1*. **(B)** Magnified view of FISH images for *PxutGr1* and *PxutOBP9*. *PxutGr1* expressed in a small cell adjacent to *PxutOBP9*-expressing large support cell.

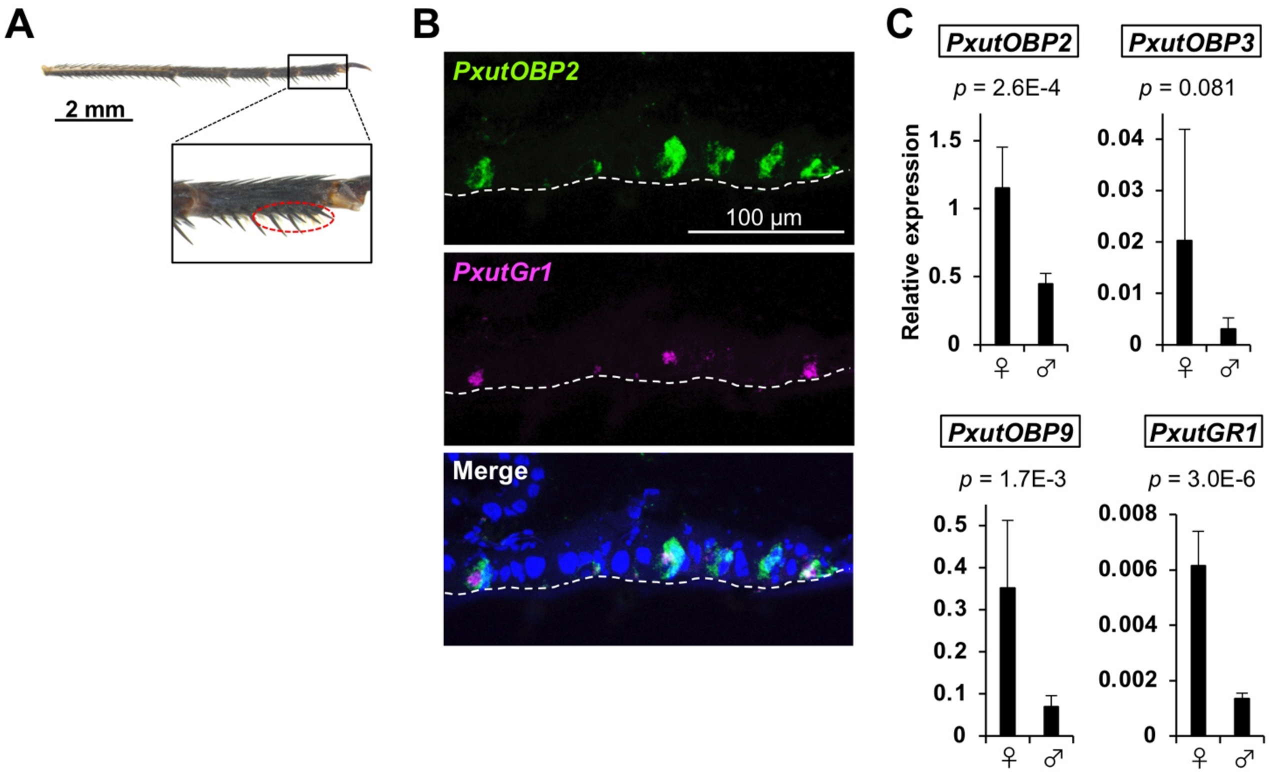


**Supplementary Figure S4.** *OBP*s expression in the male foreleg. **(A)** Male foreleg tarsus with fewer gustatory sensilla than female. **(B)** Simultaneous detection of *PxutOBP2* and *PxutGr1* expression in the male fifth tarsomere. **(C)** Comparison of expression levels of *OBP*s between male and female foreleg tarsus sampled from 0-day-old adults performed by qRT-PCR. Three individuals were used per lot. All data are shown as the means ± SD (n = 6, each sex). Student's *t*-test was conducted.

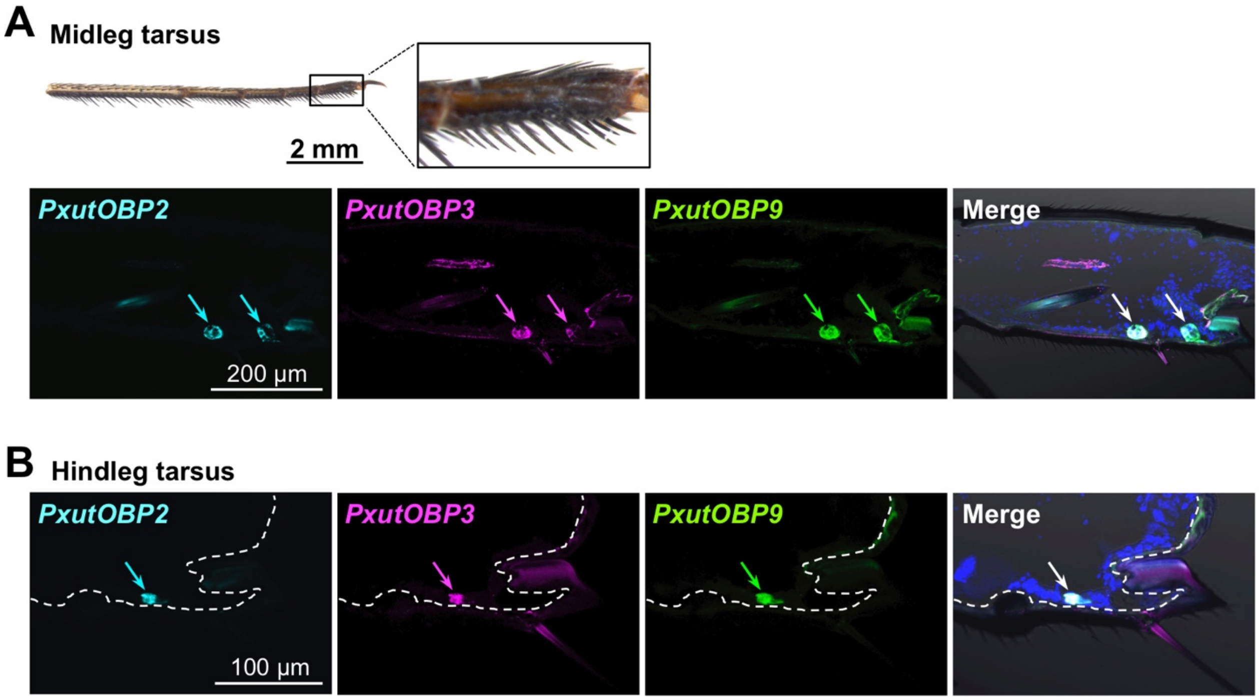


**Supplementary Figure S5.** *OBP*s expression in the female midleg and hindleg. **(A)** Female midleg tarsus with fewer gustatory sensilla. Simultaneous detection of *PxutOBP2*, *3*, and *9* expression in the female fifth tarsomere of midleg tarsus by triple-color FISH. Magnified views are shown in **Figure 4A**. **(B)** Simultaneous detection of *PxutOBP2*, *3*, and *9* expression in the female fifth tarsomere of hindleg tarsus by triple-color FISH. –1-day-old females were used.

**Supplementary Table S1.** Gene specific primers for qRT-PCR experiments.

| Gene | Forward (5' to 3') | Reverse (5' to 3') |
|---|---|---|
| *PxutRpl32* | CAATGTCCGTGAGCTTGAGA | GGCGCGCTCTACTATTGTCT |
| *PxutGr1* | AGAAGCAATGAAAGAGCGCA | ACAGCGTACACCAACATTGAG |
| *PxutOBP1* | TGACGTCGAAAAGTCTTCTGAG | CGTCACTGACTGCTTCATCG |
| *PxutOBP2* | GCGTACAAGAAGGCTGGAAT | TCATAGTGTCGGGATCGTCA |
| *PxutOBP3* | GCCACAGCGAAATCCTGTAT | TTAACAACGCCGACCTTTTC |
| *PxutOBP4* | AGTGACGACACCGGACATTT | TTGTCACATGCATCAACAGC |
| *PxutOBP5* | GCTAAGGAAGCAGCATCCAC | CTTCATCGGCGTAGTTGGTT |
| *PxutOBP6* | GACCTCCAGACTGCTCCAAG | ATTGGCATCAACCCATTTGT |
| *PxutOBP7* | GAACCTTGGGGACCACATAA | CAGATCCTCGGTAAGCAGGT |
| *PxutOBP8* | AGTCCAAGGCTGTTGAGGAA | ATCAGCTCCGACTTCACCAT |
| *PxutOBP9* | TGTCTCGCAGAAGAAAATGG | TAAATTTCCCGCCAGAACAG |
| *PxutOBP10* | GGGCTAACGATGGACATGAT | AAAACTTGTGCGACATGCAG |
| *PxutOBP11* | TTAAACGCTACACCCGATCC | TGCTTACATTGTTCGGTGTCA |
| *PxutOBP12* | TTTCGAATTGTGCAGCAAAG | GCTCAAAACCCATCTTTGGA |
| *PxutOBP13* | TCATGAAAATGGCAAACACGA | ATTGCAGCCTTTGTCGGTTC |
| *PxutOBP14* | GTACCGGGAAGGAAAAACCT | ATATGCCGTTTTCGCAATTC |
| *PxutOBP15* | TCGAGAGCTCGCTAAAGGAA | GCCATAGACACTGGCGATTT |
| *PxutOBP16* | TTCGACCTGCTCACTGACAC | TGGATCATGTCCACCATTTG |

| | | |
|---|---|---|
| *PxutOBP17* | AATTTTTCGCTCGGGACACG | TGTGAAGCCATTGCGCATTC |
| *PxutOBP18* | AAAAACGGCGATGAACAAAG | TCTGTCACACCCCTTGTCAC |
| *PxutOBP19* | CGCAAGCGTCGTCAGTTATG | AGTCCAGCCTCGTTTGAGTG |
| *PxutOBP20* | CGCCTAGTGGAGGAAGTGAA | CATGTCATCAGGCAACATGG |
| *PxutOBP21* | GCGTTTACCGGAAAGTGAAA | ATGAAGTAGCCACGCTCGTT |
| *PxutOBP22* | TCGGCAAAGATGTTGAAGAA | CACATCACGTTCCTTTGCTC |
| *PxutOBP23* | CATGCTGCAGAAGGAAACGG | TGCTCTGTCACAACCCTTTTC |
| *PxutOBP24* | GGGACAAGCGGTGAAGAATA | GCAACTCCCTTGCACTTTTC |
| *PxutOBP25* | TTTGTGCTCTGTCAGCCTTG | TGATATCCGCACCAGTTTCA |
| *PxutOBP26* | AGCGGTTATCAAGCAAGTGG | CATTTGGCTGTCCAGTACGA |
| *PxutOBP27* | TGGATGTATGGGCTGAAGCT | GCAACAGTAGCAATGCAAGAGA |
| *PxutOBP28* | GAAAAACGTTGCTAATTGCGTA | AAATTCGGTGGATCAGCTTC |
| *PxutOBP29* | GTGCCTCGCTAAAGAAATGG | TGCCAGTTAATTCGTTGCAG |
| *PxutOBP30* | AAGTCCACGCGACCTTCAAA | ACATCCCAGTTGCCTGATGT |
| *PxutOBP31* | TGACTTGTTTCGCCACTGAG | TGTCATTTGGTCGCACTGAT |
| *PxutOBP32* | TTACATTCGTTGCGTGTTGG | CACTGCAAAGCCATGTCAAT |
| *PxutOBP33* | ATGGCAGCAGCAACACATTG | AGCCAATTTCGTAGTTTGTCCT |
| *PxutOBP34* | TCGATGCTTACGAACCTCCG | AATTCGGGTCCCAGAAGCTG |
| *PxutOBP35* | GACCAGGCCAAGATACTGGA | AAGTTGAGCAGCGACTTGTG |
| *PxutOBP36* | CCGACCAGTGTCTTAGCGAA | GATGACGCCGTTCGAATTCA |

| | | |
|---|---|---|
| *PxutOBP37* | TTGCTTCGCTACAACCATTG | TGCGAAGGCTTTCTTGATTT |
| *PxutOBP38* | AAGCGGCGATAAACGAATGT | TGCCTCCATTCTTCTCTCCA |
| *PxutOBP39* | AAGTTACCGGCACAAATTGC | CGTTGCCTTCATTTTCACTG |
| *PxutOBP40* | GCCAGTCGTTGAGAGAGGAC | TCAGAGTTGCTCCCCTGTTT |
| *PxutOBP41* | TGCGCGTTCATCTGTATAGC | GAGTCGTTTTGCGGTTTGAT |
| *PxutOBP42* | CACTGCGTGCAATTGCTGTA | CTTGAACAAACTGCAGGCCG |
| *PxutOBP43* | TGCCTTCACAAATGGTTGAG | CGTTTTCAGGGAGATTCCAA |
| *PxutOBP44* | ACGATGAGGCGCTAAAGAAA | CTTGTCAACGTCGCTTGGTA |

**Supplementary Table S2.** Gene specific primers for riboprobe synthesis.

| Gene | Forward (5’ to 3’) | Reverse (5’ to 3’) |
|---|---|---|
| *PxutGr1* | CGTCGTCTTGGGAAGTTGTT | AGTACGGGGGAATGTTTGTG |
| *PxutOBP1* | AGTGGTACAGAGCTGGGAACA | GTCAGCGGCCAATGTAACTT |
| *PxutOBP2* | TGGCGCTGTTTATATTTCAAG | CGAACTCATCGGCTTTATCA |
| *PxutOBP3* | TTTGTCAAAACGTCGAAAATG | TGCAGTTCAGGAGCGTACTT |
| *PxutOBP9* | ATGGCGGCTGTGTACTTCAT | TTTTACTGCACTGAGCGTCCT |
